# The Structural E/I Balance Constrains the Early Development of Cortical Network Activity

**DOI:** 10.3389/fncel.2021.687306

**Published:** 2021-07-19

**Authors:** Wenxi Xing, Ana Dolabela de Lima, Thomas Voigt

**Affiliations:** Medizinische Fakultät, Institut für Physiologie, Otto-von-Guericke Universität, Magdeburg, Germany

**Keywords:** cerebral cortex, gamma-aminobutyric acid, cell culture, development, projection neurons, interneurons, cortical network

## Abstract

Neocortical networks have a characteristic constant ratio in the number of glutamatergic projection neurons (PN) and GABAergic interneurons (IN), and deviations in this ratio are often associated with developmental neuropathologies. Cultured networks with defined cellular content allowed us to ask if initial PN/IN ratios change the developmental population dynamics, and how different ratios impact the physiological excitatory/inhibitory (E/I) balance and the network activity development. During the first week *in vitro*, the IN content modulated PN numbers, increasing their proliferation in networks with higher IN proportions. The proportion of INs in each network set remained similar to the initial plating ratio during the 4 weeks cultivation period. Results from additional networks generated with more diverse cellular composition, including early-born GABA neurons, suggest that a GABA-dependent mechanism may decrease the survival of additional INs. A large variation of the PN/IN ratio did not change the balance between isolated spontaneous glutamatergic and GABAergic postsynaptic currents charge transfer (E/I balance) measured in PNs or INs. In contrast, the E/I balance of multisynaptic bursts reflected differences in IN content. Additionally, the spontaneous activity recorded by calcium imaging showed that higher IN ratios were associated with increased frequency of network bursts combined with a decrease of participating neurons per event. In the 4th week *in vitro*, bursting activity was stereotypically synchronized in networks with very few INs but was more desynchronized in networks with higher IN proportions. These results suggest that the E/I balance of isolated postsynaptic currents in single cells may be regulated independently of PN/IN proportions, but the network bursts E/I balance and the maturation of spontaneous network activity critically depends upon the structural PN/IN ratio.

## Introduction

Two major classes of neurons form the neocortical networks in mammals: Glutamatergic and GABAergic neurons. With some notable exceptions, glutamatergic neurons are projection neurons (PNs) and form depolarizing synapses, and GABAergic neurons are interneurons (INs) and form synapses that in early development depolarize and later hyperpolarize the postsynaptic neurons. The balance between excitatory (E) and inhibitory (I) inputs to the neurons is considered essential for the stability and function of the neocortical networks. The structural correlate of the balance may be the typical mean proportions of INs in the neocortex: in rodents 15–16% (Beaulieu, 1993; Gabbott et al., 1997), and in primates 20–25% (Hendry et al., 1987; Gabbott and Bacon, 1996; Mao et al., 2001; Froemke, 2015). Alterations of the E/I balance or IN proportions are often associated with neuropathologies, especially those with a developmental background (Ramamoorthi and Lin, 2011; Anderson and Baraban, 2012; Ansen-Wilson and Lipinski, 2017; Paterno et al., 2020). Even if it is not always clear if aberrations of the structural IN ratio lead to pathologies or are the consequence of other causes, transplants of additional INs have been proposed as a possible replacement therapeutic approach for pharmacoresistant pathologies (e.g., Sebe and Baraban, 2011). So far, many preclinical studies in, for example, epilepsy animal models showed that the transplantation of INs successfully improves seizure activity in the postnatal neocortex (Zipancic et al., 2010; Hunt et al., 2013; Hammad et al., 2015).

Different cellular mechanisms have been proposed to explain how the PN/IN ratio could be achieved during development (Buss et al., 2006; Southwell et al., 2012; Wong et al., 2018). Apart from the question of what regulatory processes are involved in setting up the structural E/I ratio within cortical circuits, it is also unclear if alterations form stable functional networks or obligatorily lead to pathological deviations. Even if the E/I balance in the functional network is dynamically and homeostatically regulated (Haider and McCormick, 2009; Isaacson and Scanziani, 2011; Froemke, 2015), the question remains if the structural development constrains the dynamic regulatory ranges (see for example, He and Cline, 2019).

In this article, we investigate the development of neuronal networks that start with different predefined PN/IN ratios. To do so we prepared neuronal cell cultures from the dorsal portion of the embryonic rat cerebral cortex at gestational day 16 (E16). At this early age, dorsal cortex (dCtx) neuroblasts generate the first projection neurons destined for layers 6 and 5. Due to the developmental gradients from rostral to caudal and medial to lateral, INs originating in the Medial Ganglionic Eminence (MGE) invade, at E16, the lower lateral portion of the rat cortical anlage, while the dorsal portion is still free of INs (Bellion et al., 2003; Bellion and Metin, 2005; Batista-Brito and Fishell, 2009; Chu and Anderson, 2015). As a result, dCtx neurons, which are dissociated from E16 rat embryos, form networks that consist predominantly of cortical PNs. To these IN-depleted cultures, we added Venus expressing INs obtained from the MGE (MGE-INs) of E14 transgenic VGAT-Venus rat embryos in predefined ratios of low, medium and, high IN density. We then investigated the development of PN and IN populations in the different network types over a 1-month period. During this time the absolute numbers of neurons changed, but the relative proportion of INs remained constant within each set of dCtx networks. Our results also showed that after synaptic maturation both INs and PNs keep their PSCs E/I ratio constant over time (2nd–4th weeks *in vitro*) irrespective of the PN/IN population ratio variation. The calcium imaging analysis shows that although all investigated network types become active, more complex activity patterns develop only if sufficient interneurons are present.

## Materials and Methods

### Cell Culture

All experiments were carried out according to the EU directive 63/10 EU. Dissociated cell cultures were prepared from embryos of time-pregnant Wistar rats and cultivated for up to 30 days in a serum-free N2 medium (75% DMEM, 25% Ham’s F12, and N2 supplements from Thermo Fisher Scientific) in the presence of a surrounding glial feeding layer. Purified astroglia cells prepared from cerebral hemispheres of newborn Wistar rats were plated at a density of 300 cells/mm^2^ in the outer portion of the Petri dish bottom 5 days before the neurons plating, as reported in detail in De Lima and Voigt (1999). Cortical neurons grew on acid-cleaned coverslips fitted to a 10 mm hole in the bottom of a 60 mm Petri dish and treated overnight with poly-D-lysine (PDL, 0.1 mg/ml in borate buffer pH 8.5, 36°C). Cultures were maintained in humidified 5% CO_2_ air atmosphere at 36°C. Three times a week the culture medium was replenished with a glia-conditioned medium by changing one-third of the total volume. In addition to the astrocytic feeder layer grown outside the neuronal network, freshly dissociated astrocytes were added to all cultures between 5 and 8 DIV. Adding astrocytes to neuronal networks toward the end of the 1st week decreased the tendency to cell aggregation and network detachment. As a consequence, healthy neuronal networks could be grown routinely with the same high quality over the entire 4-week period.

The cortical neurons were dissected from E16 time pregnant Wistar rat embryos (day after insemination = E1) in two sets with different cellular content. In one set, only the dorsal portion of the E16 cortex was dissected (dCtx), while in an additional smaller set the entire dorsolateral cortical wall was taken, excluding hippocampus and basal telencephalic anlage (wCtx). At this age, due to the developmental gradients in the cortical anlage from lateral to medial, the dorsal portion is developmentally younger and more immature than the lateral portion (Bellion et al., 2003). GABAergic precursor cells originating from the MGE (MGE-INs) migrate into the ventrolateral portion of the developing cortex, while the dorsomedial part is still free of INs. In both sets of experiments, the MGE was prepared from E14 embryos (see below) and dissociation did not differ among experimental sets.

### MGE Networks

GABAergic precursor cells were obtained from the MGE of E14 time pregnant homozygote Wistar rat embryos that express the eGFP derivate Venus under the vesicular GABA transporter (Uematsu et al., 2008). Venus expression was verified before MGE dissection by inspecting each embryo under the fluorescence microscope. Only embryos expressing Venus in the CNS were used for preparation. As E14 MGE in rat embryos contains a high density of GABA precursor neurons destined for the cerebral cortex, this preparation provided a highly enriched fraction of cortical INs. To characterize this cell population in more detail the dissociated MGE cell fraction was plated with 1,500 cells/mm^2^ and cultivated under the same conditions as all other cortical networks. The 4 h live counts confirmed that 84.5% of the dissociated cells had successfully attached to the coverslips (1,268.1 ± 34.7 cells/mm^2^; *n* = 74 fields; two randomly chosen fields per randomly picked culture; three experiments). Twenty-four hours later, cell density increased by a factor of 1.37 (1,738.0 ± 39.8 cells/mm^2^; *n* = 74 fields). At 7 DIV neuronal density was 970.8 ± 49.9 NeuN positive cells/mm^2^ (*n* = 40 fields, four cultures, two experiments). The majority of neurons were INs, co-expressing NeuN and Venus (90.6% ± 0.96; *n* = 40 fields, four cultures, two experiments). For the long-term development of neuronal populations in MGE networks see “Results” section.

### Cortical Networks

To obtain IN-depleted neuronal networks (T00 networks), dissociated cells obtained from the IN-free dCtx of E16 wild-type rat embryos were plated with a density of 1,500 cells/mm^2^ onto Poly-D-Lysine treated coverslips. The live counts 4 h after plating confirmed that 74% of the plated cells attached successfully to the coverslip (1,101.0 ± 18.3 cells/mm^2^; *n* = 270 fields; two randomly chosen fields per randomly picked culture; 15 cultures per preparation, total nine experiments). Ongoing cell proliferation increased this density within 24 h by a factor of 1.47 to about 1,614.0 ± 30.8 cells/mm^2^ (*n* = 270). Due to the stop of cell proliferation by Ara-C after at 2 DIV, and by the natural occurrence of cell death, neuronal density dropped afterward. At 7 DIV the T00 networks had a mean neuron density of 1,309.7 ± 42.5 NeuN positive neurons/mm^2^ (*n* = 180 fields, 15 cultures, six experiments). Only very few neurons in T00 networks were immunolabeled by anti-GABA antibodies (0.8 ± 0.25 INs/mm^2^; *n =* 180; 15 cultures; six experiments). These GABA neurons are the first to enter the developing cortex (Voigt et al., 2001) and have been described in cortical cell cultures as parvalbumin-expressing L-GABA neurons (De Lima and Voigt, 1997; Voigt et al., 2001; De Lima et al., 2004).

### Cortical-MGE Networks

To obtain networks with defined PN/IN ratios, the intended plating density of the E16 wild-type networks was verified by live cell counts at 4 h after plating. Under phase contrast illumination, all attached cells were counted in two randomly chosen fields of 15 randomly picked cultures. Twenty-four hours later the cell density of the wild-type network was assessed again (live cell counts at 1 DIV). Based on the acute density determined for each experiment in the 24 h live count the desired cell density of the freshly dissociated E14 MGE cells was plated onto the wild-type network to obtain the intended PN/IN cell ratio. Three types of networks were generated as follows: **T05** networks, with five cells of MGE origin added to every 100 neocortical cells (5:100, 4.8% MGE cells in total cell density); **T25** networks, with 20–40 MGE cells added to every 100 neocortical cells (30:100; 23.1%); and **T45** networks, with 80 MGE cells added to every 100 neocortical cells (80:100; 44.4%). T00 networks were cultivated in all preparations as controls for intrinsic IN content (see above).

After adding the appropriate amount of MGE cells both cell populations were allowed to proliferate for another 24 h before cell proliferation was stopped by application of the mitotic inhibitor 1-ß-D-Arabinofurano-sylcytosine (Ara-C; 5 μM final concentration; Calbiochem). Ara-C treatment was ended by a complete exchange of fresh glia conditioned N2 medium. According to this time schedule, cortical precursor cells could proliferate for 48 h and MGE precursor cells for 24 h after plating. The Ara-C treatment was required to stop the otherwise uncontrolled cell proliferation. In this way, all cultures were set to the same starting conditions and their post-mitotic development could be compared between different network types. During the 4-week cultivation period, all network types received exactly the same treatment.

### Immunocytochemistry

**Figure 1:**
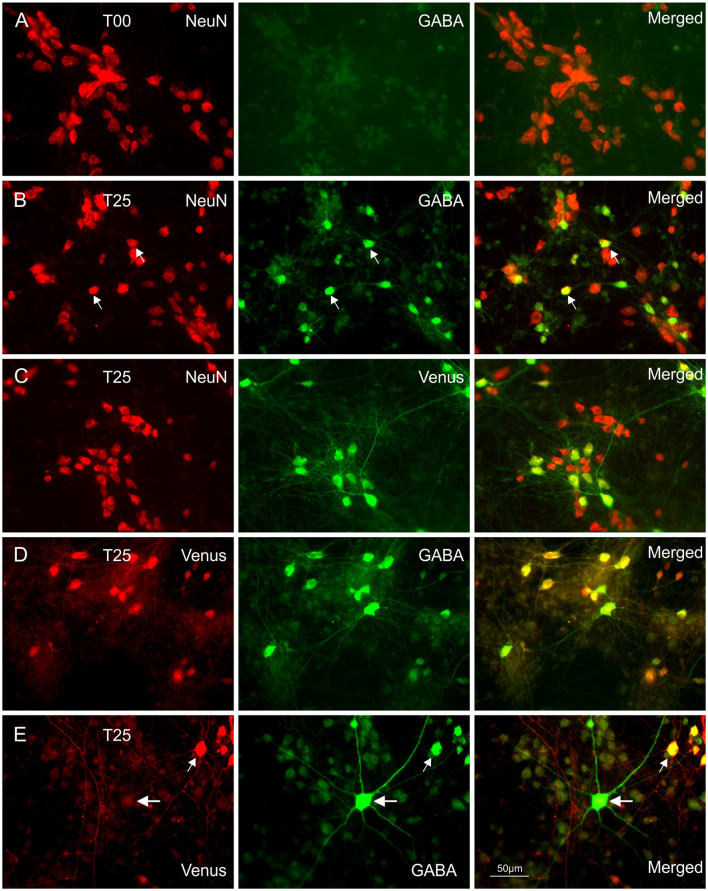
Immunocytochemical double staining for co-localization of NeuN with GABA or Venus. Images in **(A)** show a NeuN/GABA double-stained culture obtained from dissociated embryonic rat wild-type dorsal cortices (dCtx) at E16 (T00 network). In this network type, the vast majority of neurons were pyramidal neurons (PNs). Images in **(B)** show a NeuN/GABA double-stained wild-type dCtx with 25% Venus MGE-INs (T25: MGE-INs from transgenic vGAT Venus rats). Arrows point to two strongly positive NeuN/GABA neurons. **(C)** Images of a NeuN/Venus double-stained T25 culture show the vGAT-Venus expressing MGE-INs. **(D)** Images of a double-stained T25 culture show the GABA co-localization in Venus expressing neurons. **(E)** Images of a Venus/GABA double-stained T25 culture show one example of the rare Venus negative INs found in very low density in dCtx cultures (large arrows). The double-labeled Venus/GABA expressing neurons (small arrows) were of MGE origin (MGE-INs) from E14 transgenic animals. All images in **(A–E)** show 14-day-old cultures.

Co-localization of different antibodies (see Table 1) was used to identify and analyze the development of different neuron populations, including also cell death and cell proliferation. Routinely 3 to 4 fluorescent cell markers were combined. In all cases, antibody incubation was done consecutively at RT in complete sets consisting of the first antibody incubation overnight, followed by the appropriate secondary antibody for 2 h. Staining sets were separated by 30 min paraformaldehyde fixation. The consecutive scheme yielded much better results compared with simultaneous application of antibody cocktails (mixture of all primary antibodies followed by a mixture of all secondary antibodies). Thorough washing with 3 × 5 min PBS between each step was essential for good staining quality. Before embedding the coverslips in Fluoromount, they were briefly treated with DAPI to label the cell nuclei. To show MGE Venus expressing neurons we used e-GFP antibodies (Table 1). Wild-type networks (T00) were cultivated in every preparation to control for GABA cell content in the receiving cortical networks. Since no MGE cells were added, GABA neurons were stained with anti-GABA antibodies followed by anti-NeuN and DAPI staining (Figure 1A).

**Table 1:** Antibodies used in this study.

Antibody	Source	Final concentration
Monoclonal mouse anti-NeuN	Millipore, clone A60; #MAB377	1:1,000
Rabbit anti-GABA	Millipore, AB131-rb, polyclonal antibody	1:2,000
Chicken anti-eGFP	Aves, #GFP-1020	1:2,000
Monoclonal mouse anti-BrdU	Sigma, #11170376001	1:50
Polyclonal rabbit anti-Caspase 3	Asp175, Cell signaling Technologies, # 9661	1:250
Rat anti-Somatostatin	Millipore, #MAB354	1:100
Mouse anti-Parvalbumin	Swant, #PV235	1:10,000
Mouse anti-Calretinin	Swant, #6B3	1:2,000
Alexa Fluor 488 goat anti rabbit IgG	Molecular Probes #A-11008	1:250
Alexa Fluor 488 goat anti chicken IgG	Molecular Probes #A-11039	1:250
Alexa Fluor 555 goat anti-mouse IgG	Molecular Probes, A-21422	1:250
Alexa Fluor 555 goat anti-rat IgG	Molecular Probes, A214342	1:250
Cy5-goat anti-rabbit IgG	Dianova, #111–175–144	1:400

*Source and dilutions of antibodies used for immunocytochemistry*.

### Cell Density Analysis

The data acquisition of immunolabeled cultures was done blindly after assigning randomized numbers to each culture directly after staining. Each experiment typically consisted of 48 cultures with three cultures for each of the four network types and four ages. When the total density of dissociated cells was too low to plate all dishes as planned, the number of cultures was adjusted accordingly, and the experiment was repeated with an identical design. To assess cell densities, photomicrographs were taken from 10 randomly chosen fields per culture with the appropriate filter sets and 40× lens. For the analysis of caspase labeled neurons, a 100× oil immersion lens was used and the number of analyzed fields was increased accordingly. For cell density analysis, *n*-values are the numbers of analyzed fields/images. These fields were distributed evenly over the coverslip so that the entire cultivation area was sampled. Depending on the number of fluorochromes used, 3 to 4 micrographs were taken from each field with the appropriate filter settings. These micrographs were combined to one RGB image with the MetaMorph software (version v.7.8.0.0; Molecular Devices, LLC). For cell density acquisition, immuno-positive cells were counted manually, aided by MetaMorph. Cells labeled Venus(+)/NeuN(+) or, in case of T00, GABA(+)/NeuN(+) were counted as INs and cells labeled Venus(−)/NeuN(+) were counted as PNs. For all other analyses, gray values of region of interest (ROIs) were logged to data files. All acquired data were further processed with MATLAB (MathWorks Inc.). The number of experiments for the cell density series was as follows: dCtx: 9; high/low density: 5; wCtx: 3.

### Characterization of the MGE-INs

In the transgenic rats used in this study the fluorescent marker, Venus was co-expressed with vesicular GABA uptake transporter (VGAT; Uematsu et al., 2008). Co-expression of anti-GABA and anti-eGFP (Venus) was verified at 14 DIV in T25 type cultures from two preparations (e.g., Figure 1D). In 56 pairs of photomicrographs taken with a 40× epifluorescence lens, 369 Venus(+) cells from MGE origin were marked. After transferring the ROIs from the Venus image to the corresponding GABA micrograph, 352 cells were identified as INs, based on the criteria that the mean ROI pixel intensity was larger than five times the standard deviation of the mean gray values measured in three background ROIs positioned in a cell-free region. Thus, 95.4% of the Venus labeled cells expressed sufficient fluorochrome to identify them unambiguously as GABAergic. Based on this result, an Venus(+)/NeuN(+) neuron from transgenic Venus rats will be addressed throughout this article as IN and Venus(−)/NeuN(+) neuron as PN.

**Figure 2:**
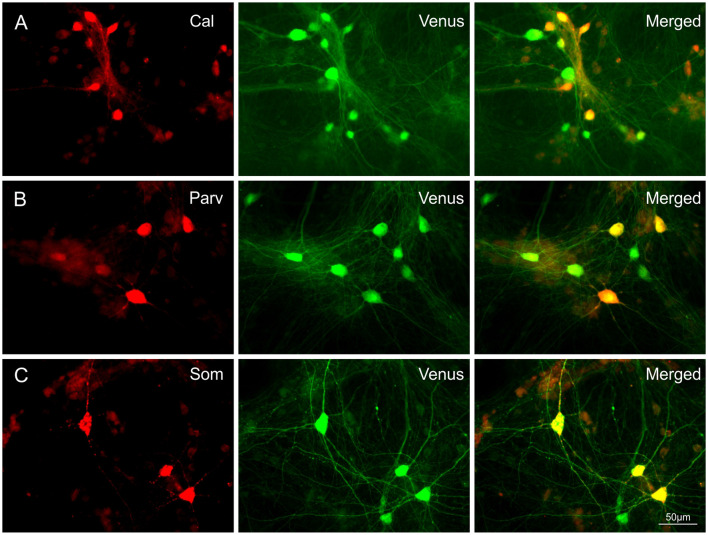
Co-expression of Calretinin (Cal), Parvalbumin (Parv), or Somatostatin (Som) in Venus expressing neurons. Images show the co-localization of Cal **(A)**, Parv **(B)** or Som **(C)** with Venus in neurons of 28-day-old T25 networks. Differences in soma size between Figures 1 and 2 are due to age differences of the cultures at the time of fixation.

To assess the occurrence of IN subtypes, Venus neurons were immunolabelled with GFP and calretinin (CR), parvalbumin (PV), or somatostatin (SOM) antibodies in 28-day-old T25 dCtx/MGE cultures from two preparations (Figure 2). These experiments confirmed that all three markers for cortical INs with MGE origin were also found *in vitro* in the cortex/MGE networks as follows: CR 13.4% [389 out of 2,899 Venus(+) cells, 12 cultures]; SOM 17.3% [406 out of 2,342 Venus(+) cells, 12 cultures]; PV 6.9% [165 out of 2,383 Venus(+) cells, 14 cultures]. Note that the content of PV-, SOM-, and CR-expressing neurons in culture were not expected to match the adult rat cortex expression, as the dissociation of MGE neurons at E14 captures only a fraction of all the future INs that normally migrate to the neocortex.

### Caspase Immunocytochemistry

To assess apoptotic cell death between 2 and 6 DIV, networks were stained on a day-by-day basis (two experiments, three cultures per day and experiment) with anti-NeuN (red), anti-eGFP (Venus) or anti-GABA (T00; green), anti-Caspase (infra-red), and DAPI (blue; Antibodies information in Table 1). Five micrographs with the appropriate filter setting were taken from randomly chosen fields with 100× oil objective. After positioning ROIs onto PN neurons, the mean gray values of the corresponding caspase images were logged along with three background ROIs positioned in cell-free regions. After background correction, the intensity of the anti-Caspase staining was determined in all PN neurons in MATLAB with a predefined threshold.

### BrdU Immunocytochemistry

Cell proliferation was assessed by using the proliferation marker 5-Bromo-2′-deoxyuridine (BrdU; 2 μM final concentration; SERVA #15240) at 2 DIV, and by omitting the application of Ara-C. Cultures were immunolabeled with anti-BrdU, anti-eGFP (Venus), and DAPI after 12 h or 24 h BrdU incubation times. Cells were analyzed in photomicrographs taken with 40x at the appropriate filter setting. IN and PN neurons were analyzed by positioning a ROIs in the corresponding images over the soma. ROIs were then transferred to the 12-bit gray values of the BrdU images. A neuron was counted as BrdU(+) when its gray value exceeded the median gray values obtained from three BrdU(−) and three weakly BrdU(+) labeled neurons selected by the observer. Data were sampled from 100 images (range: 99–117) from a total of 12 cultures per network set (T00, T05, T25, T45; three cultures per set from each of the four independent experiments). The number of individually analyzed neurons in different network types was as follows. T00: 9,388 PNs; T05: 9,715 PNs and 1,485 INs; T25: 10,320 PNs and 421 INs; T45: 10,716 PNs and 736 INs.

### Soma Size Measurement

For soma size analysis of GABA and non-GABA neurons T05, T20, and T30 type cultures from four independent preparations were stained for anti-eGFP (Venus), anti-NeuN, and DAPI. Cells were analyzed in photomicrographs taken with 40× at the appropriate filter setting. The soma surface of a total of 3,162 INs and 2,447 PNs was calculated with MetaMorph from hand-drawn soma outlines. The number of analyzed cells for 7, 14, 21 DIV in Figure 4J were as follows: IN T05 (327, 286, 300); IN T20 (374, 303, 368); IN T30 (415, 340, 449) and for 21 DIV in Figure 4K: PN T05 (210); PN T20 (175); PN T30 (203).

**Figure 3:**
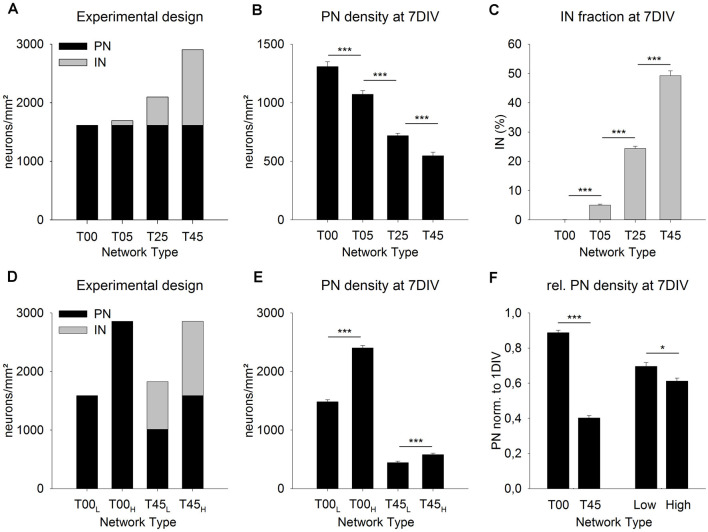
MGE-INs modulate the density of PNs during the 1st week *in vitro*. Networks with increasing MGE-IN content were generated by plating dissociated MGE-INs (**A**, gray bars, 1 DIV) on cortical networks with the same density of PNs (**A**, black bars). After 7 DIV the projection neurons (PN) density was highest in T00 and lowest in T45 networks **(B)**. The PN/IN ratio did not change during the 1st week *in vitro* **(C)**. **(D)** Network pairs with no MGE-INs (T00) or high MGE-IN content (T45) were generated with low (L) or high (H) total cell density. **(E)** The much higher loss of PNs in T45_L_ and T45_H_ networks showed that PN density during the 1st week was predominantly dependent on IN density rather than on total cell density. **(F)** For statistical comparison, the data was normalized to 1 DIV values and pooled in four groups (see “Results” Section). This analysis showed that the presence of INs (T00 vs. T45) had a larger effect on PN density decline than total cell density (Low vs. High). Asterisks show the level of statistical significance (*P ≤ 0.05, ***P ≤ 0.001).

**Figure 4:**
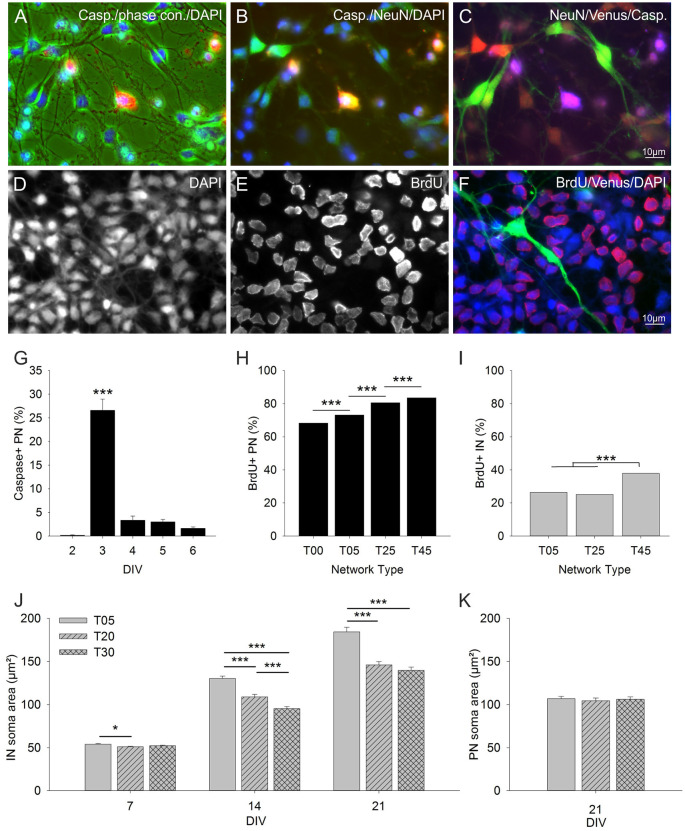
Influence of IN density on PN cell death and cell proliferation rates. The images in **(A–C)** show three RGB channel combinations of the five black and white micrographs taken with different illumination from an exemplary field of an anti-Caspase stained T25 network at 3 DIV (quantitative data in graph **G**). **(A)** caspase (red), phase contrast (green), DAPI (blue). **(B)** Caspase (red), NeuN (green), DAPI (blue). **(C)** NeuN (red), Venus (green), caspase (blue). Panels **(D–F)** show an exemplary field of a BrdU staining in a T25 network 24 h after BrdU application (quantitative data in graphs **H** and **I**). **(D)** DAPI, **(E)** BrdU, **(F)** color combined BrdU (red), Venus (green), DAPI (blue). **(G)** Graph shows the fraction of anti-Caspase positive PNs in T25 networks between 2–6 DIV. The number of caspase positive PNs increased drastically 24 h after the addition of the mitotic inhibitor Ara-C at 2 DIV and dropped to low values afterward. **(H)** Fraction of anti-BrdU positive PNs in different network types between 2–3 DIV (standard AraC treatment was replaced by BrdU at 2 DIV and proliferation rates were assessed 12 and 24 h later; see “Materials and Methods” section). The number of proliferating PNs increased with increasing IN content. **(I)** Fraction of anti-BrdU positive INs. Only T45 networks showed a significant increase in IN proliferation rates. Panels **(J,K)** show the analysis of IN and PN soma size in T05, T20, and T30 networks. **(J)** IN soma size was largest in networks with the lowest IN density (T05) and decreased with increasing IN density (T20, T30). PNs soma size did not vary with IN content **(K)**. Asterisks show the level of statistical significance (*P ≤ 0.05, ***P ≤ 0.001). For *n* values see “Materials and Methods”, for statistics, see “Results” Section.

### Electrophysiology

For patch-clamp recording, an acrylic insert was positioned into the culture dish to make a recording chamber with a volume of 1–1.5 ml. The heat-controlled chamber (Inline Heater SH-27B, TC-324B; Warner Instrument Corporation) was mounted on the stage of an inverted microscope (ZEISS Axiovert S100 TV) and constantly perfused with a HEPES-buffered artificial cerebrospinal fluid (aCSF; ionic composition in mM: 140 NaCl, 5 KCl, 3 CaCl_2_, 1.5 MgCl_2_, 1.25 NaH_2_PO_4_, 20 D-glucose, and 15 HEPES/NaOH, pH 7.4) at 1–2 ml/min. Patch pipettes were pulled from borosilicate glass (GC150TF-10, Harvard Apparatus LTD, London, UK) with tip resistances of 2.5–4.5 MΩ. For voltage-clamp experiments pipettes were filled with a solution containing [in mM]: 117 D-gluconic acid, 13 CsCl, 1 MgCl_2_, 0.07 CaCl_2_, 10 HEPES, 0.1 EGTA, 4.5 Mg-ATP, 0.75 Na-GTP. D-gluconic acid was titrated with CsOH (pH 7.2; 290–300 mOsmol/kg) to pH 7.25.

INs and PNs were identified under brief UV illumination according to their Venus expression using a 40× fluorescence objective. Whole-cell voltage-clamp recordings were done at 27–29°C with a patch-clamp L/M-EPC-7 amplifier (List-Medical-Electronic). Data acquisition (filtered at 1kHz) was performed with Pulse software (v.8.74, HEKA Elektronik Dr. Schulze GmbH). Liquid junction potential was corrected for 10mV. Immediately after the establishment of the whole-cell configuration in voltage-clamp mode, the resting membrane potential was measured. Spontaneous glutamatergic postsynaptic currents (EPSCs) were recorded at a holding potential of −60 mV as inwardly directed currents. After 5 min recording time, the holding potential was changed to 0 mV and spontaneous GABAergic postsynaptic currents (IPSCs) were recorded for another 5 min as outwardly directed currents. The polarity switch sequence was altered randomly between recordings. In control experiments, the nature of glutamatergic PSCs was confirmed by a block of spontaneous activity with 6-cyano-7-nitroquinoxaline-2,3-dione disodium salt (CNQX, 2.5 μM) and D-2-amino-5-posphonopentanoic acid (APV, 12.5 μM; Tocris, Bristol, UK) at a holding potential of −60 mV (*n* = 12). The nature of GABAergic PSCs was confirmed by a block of spontaneous activity at a holding potential of 0 mV with (−)-bicuculline methiodide (BMI, 10 μM, *n* = 21) or SR 95531 (gabazine, 25 μM; Tocris, Bristol, UK, *n* = 8). All drugs were added to the bathing solution.

Synaptic currents were analyzed with MiniAnalysis software (version 6.0.3, Synaptosoft, Decatur, GA, USA). Isolated PSCs (Figures 8A,B and arrows in **D**) were selected by hand in the MiniAnalysis software. For analysis, the minimal requirement was ≥30 well-isolated sEPSCs and ≥30 sIPSCs. The maximal number was set to 100 EPSCs and 100 IPSCs per cell. In T00 networks, a cell with 0 IPSCs and ≥30 EPSCs per 10 min total recording time was categorized as free of GABA inputs. Mean charge transfer of PSCs was obtained from integral area values (pAms) in MiniAnalysis. The mean charge transfer of large network bursts (Figure 8) was analyzed in MATLAB by calculating the integral area values above baseline. The beginning and end of each event were selected by hand. A total of 218 PNs and 229 INs were recorded in T00 (*n* = 51 PNs), T05 (89 PNs, 113 INs), and T45 (78 PNs, 116 INs) networks between 6 DIV and 30 DIV (188 cultures from six independent preparations). For statistical analysis PSC recordings of the same cell type and network type were grouped together as follows: 1st week (6–9 DIV, 15 PNs, and 29 INs), 2nd week (12–16 DIV, 61 PNs, and 74 INs), 3rd week (19–23 DIV, 71 PNs, and 61 INs), 4th week (26–30 DIV, 71 PNs, and 65 INs).

**Figure 5:**
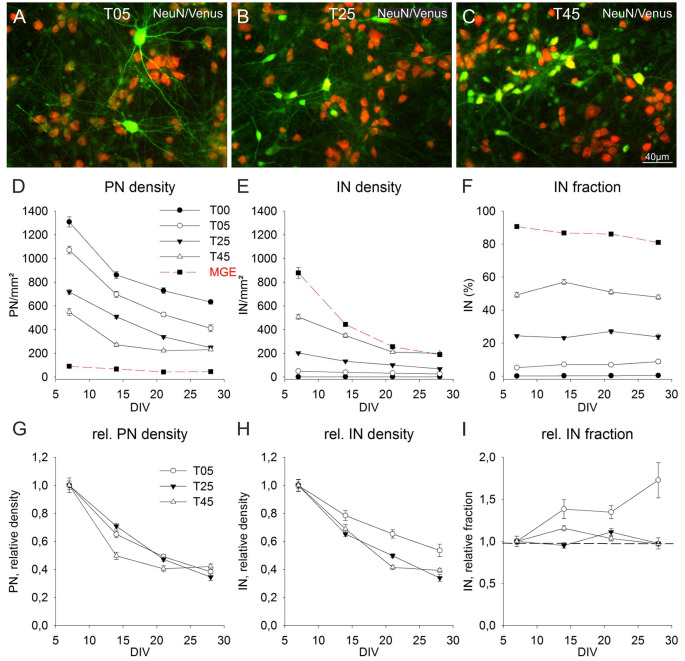
Long-term population dynamics in different network types. Panels **(A–C)** show the color combined images of the Venus (green) and NeuN (red) staining in exemplary fields of 14-day-old T05 **(A)**, T25 **(B)**, and T45 **(C)** dCtx networks. The networks differ in their Venus neuron density. The quantitative analysis of the apparent soma size differences of Venus neurons between the different network types is shown in Figure 4 **(J,K)**. **(D–F)** Graphs show the development of PN **(D)** and IN **(E)** densities, and the fraction of INs **(F)** between 7 and 28 DIV. For better comparison, the data of T05, T25, and T45 networks were normalized to 7 DIV and are shown in **(G–I)**. The dotted line in graph **(I)** shows the normalized 7 DIV value for reference. For MGE networks in (**D**, red dashed line) non-GABA neuron density is shown instead of the PN density.

**Figure 6:**
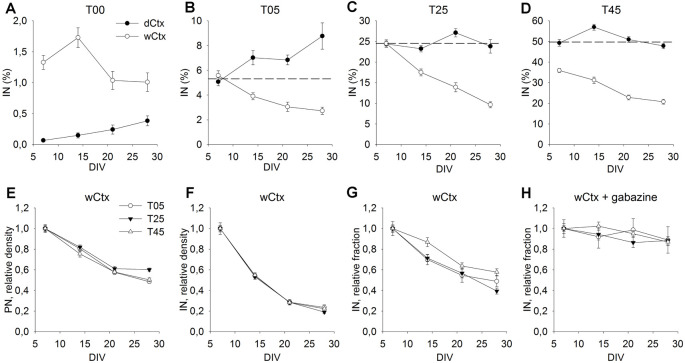
Cellular composition of dCtx and wCtx networks during maturation. The graph in **(A)** shows the MGE-IN fraction of T00 networks derived either from the dorsal cortex (dCtx) or whole cortex (wCtx) of wild-type E16 rat embryos. In **(B–D)** the MGE-INs fraction of dCtx and wCtx networks is shown for T05 **(B)**, T25 **(C)**, and T45 **(D)** cultures. On wCtx the fraction of MGE-INs declined with age in all cases, while on dCtx it increased in T05, but remained stable in T25, T45 network types. **(E–H)** The values normalized to 7 DIV allow to compare the population dynamics of PN **(E)**, IN **(F)**, and %IN **(G)** in wCtx T05, T25, and T45 cultures. The faster decline in the IN fraction **(E–G)** was abolished in the presence of the GABA antagonist Gabazine **(H)**. Dotted lines in **(B–D)** show the normalized 7 DIV value for reference.

**Figure 7:**
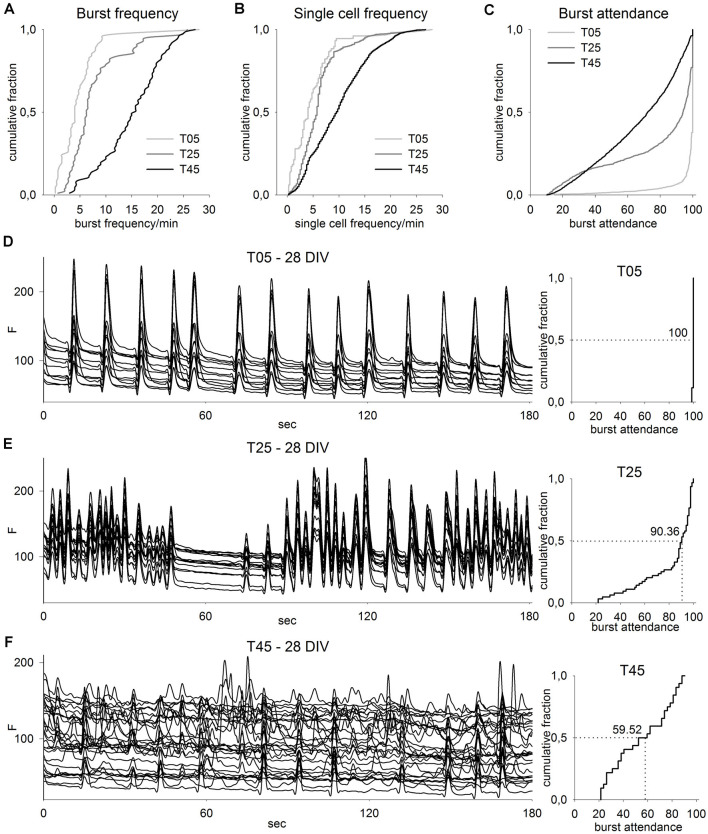
Calcium imaging analysis of different network types. **(A–C)** CDF graphs show the imaging results of T05, T25, and T45 network recordings. With increasing MGE-IN content, network burst frequency **(A)** and single-cell burst frequency **(B)** increased but burst attendance **(C)** decreased (T05: *n* = 82, T25: *n* = 90, T45: *n* = 87; numbers correspond to the sum of data from 10 recorded fields in each of three cultures between ages 14 and 28 DIV from each of three independent experiments). **(D–F)** Imaging traces from exemplary fields of T05 **(D)**, T25 **(E)**, and T45 **(F)** networks (left, 28 DIV) show for clarity only 3 min of the recording time and a reduced number of traces. The corresponding CDF plots of burst attendance (right) are calculated from the background-corrected dF/F_0_ values (see “Materials and Methods” section) and include the data of the entire 4 min record and all active neurons within the fields. The median of the burst attendance (number of neurons participating in a burst) is shown in each plot.

**Figure 8:**
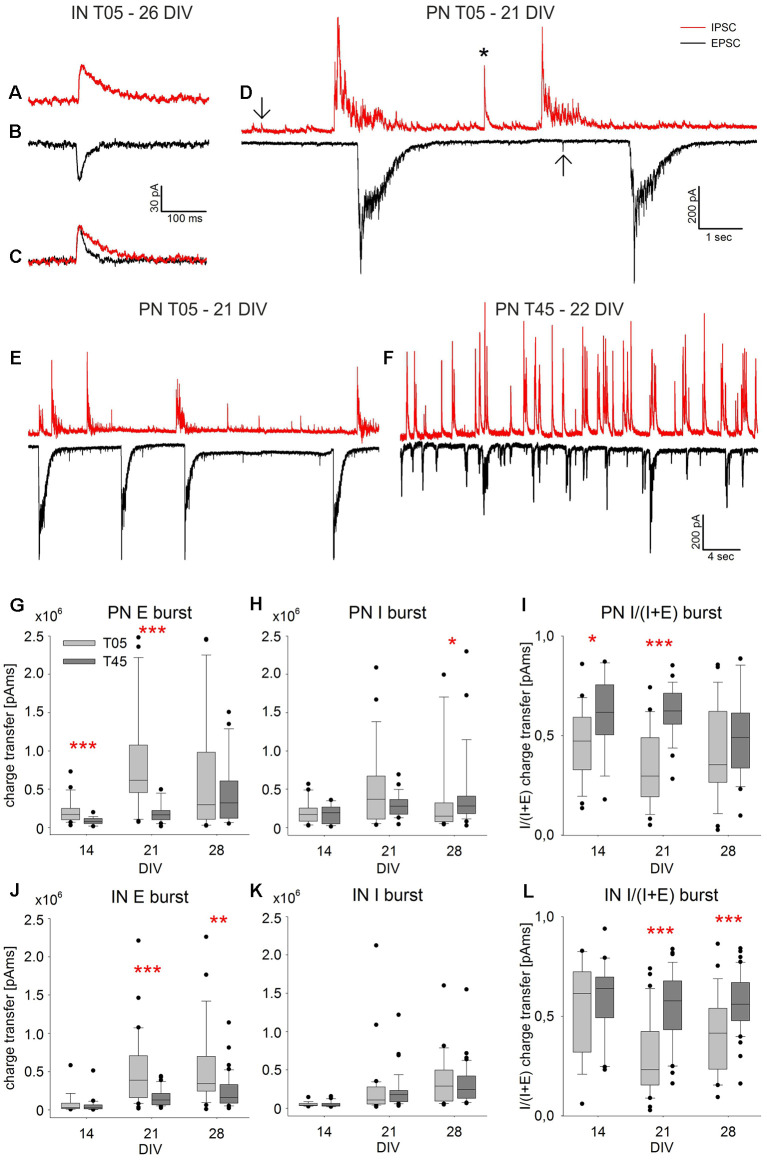
Spontaneous PSC Bursts. The exemplary spontaneous IPSCs **(A)** and EPSCs **(B)** were recorded in an IN (T05, 26 DIV). The slower decay time of inhibitory currents is visualized by scaling the PSCs shown in **(A,B)** to the same amplitude **(C)**. The examples of single isolated PSCs (**D**, arrows), a short multisynaptic event (**D**, asterisk), and large network bursts **(D)** were recorded in a PN (T05, 21 DIV). While isolated PSCs are single synaptic events (**A–C**, arrows in **D**), network bursts of large amplitude **(D)** are elicited by a barrage of synaptic inputs over several 100 ms. Panels **(E,F)** show exemplary traces illustrating the difference between spontaneous burst currents in PNs of T05 (**E**, 21 DIV) and T45 networks (**F**, 22 DIV). The quantitative analysis of network burst charge transfer is shown in **(G–L)**. For both neuron types, EPSC burst charge transfer increased in the 3rd week, most prominently in T05 **(G–J)**, while the increase of IPSC burst charge transfer was less pronounced **(H–K)**. As a consequence, the burst charge transfer ratio [I/(I + E)] was lower in T05 compared with T45 networks **(I,L)** reflecting the lower synaptic inhibitory drive in both neuron types of T05 networks (see also: **E,F**). Asterisks show the level of statistical significance of differences between network types (*P ≤ 0.05, **P ≤ 0.01, ***P ≤ 0.001). For statistical significance of age differences, see text.

### Calcium Imaging

Spontaneous network activity was recorded with calcium imaging at 14, 21, and 28 DIV. In each of the three experiments, we examined three cultures for each network type (T05, T25, and T45). Cultures were incubated in 5 μM Fluo-4 AM (#F14201 Molecular Probes, Thermo Fisher Scientific) for 60 min followed by several washes with aCSF. After changing the culture medium to aCSF medium culture dishes were transferred to an inverted microscope (Axiovert S100 TV, Zeiss, Oberkochen, Germany) equipped with a charge-coupled device camera (Cool Snap ES, Visitron Systems, Puchheim, Germany). The recordings were made in 10 randomly chosen fields per culture using a 20× Lens. In each field, a time-lapse series of fluorescence images were recorded at 1 Hz during 4 min sessions with MetaMorph software as described earlier (Opitz et al., 2002). A differential interference contrast (DIC) image of each field was also acquired for later cell identification. To recognize changes in the fluorescence, each frame was subtracted from the preceding one. The resulting differential images enabled us to identify regions of interest that corresponded to active neurons as confirmed with the help of DIC images. Average gray values of regions of interest were calculated and stored as log files. A change in ([Ca^2+^]_i_) was considered significant when the absolute difference of gray values exceeded five times the SD of background noise measured in cell-free areas. A burst was defined when more than 10% of all active neurons within a given field were simultaneously active. To qualify for a burst the minimal required number of coactive neurons was set to seven. Changes in calcium concentration ([Ca^2+^]_i_) were further analyzed with MATLAB.

### Statistical Analysis

All statistical tests were done with SigmaStat version 3.5 (SPSS Inc., Chicago, IL, USA). After initial tests for data normality (Kolmogorov-Smirnov-test) and Equal Variance, parametric or non-parametric statistical methods were executed. For comparison of multiple ages or culturing conditions, we used one-way analysis of variance (ANOVA), followed by the Holm-Sidak method for pairwise multiple comparisons, or Kruskal-Wallis one-way ANOVA on ranks (KW-ANOVA), followed by the Dunn’s method for pairwise multiple comparisons. To test for differences between network types, independent of age or density variations, we also used Two-way ANOVA, for factors of age or density and network type. Differences between experimental sets were also tested with *t*-test or Mann-Whitney rank sum test (MW-RST). The proportions of BrdU labeled IN and PN neurons in different conditions were compared with the chi-square-test. A *P*-value of ≤ 0.05 was considered statistically significant. Asterisks in all graphs show the level of statistical significance (*P ≤ 0.05, **P ≤ 0.01, ^***^P ≤ 0.001).

## Results

### Early Development of MGE-dCtx Networks

In the first set of experiments, we asked if the fraction of INs added to the 1-day-old dCtx network have an impact on the initial development of the network (Figure 3). Based on our experimental design, for all network types (T00, T05, T25, T45, see “Materials and Methods” section), the cortical neurons (PNs) were plated first and with the same density. One day later, increasing fractions of MGE-INs were added to groups of cultures to generate different network sets (1 DIV: T00: 0%, T05: 4.8%, T25: 23.1%, T45: 44.4%; Figure 3A, gray bars). At 7 DIV, the difference in IN proportions of the initial platings was maintained (0.07% for T00 networks, 5.08% for T05, 24.39% for T25, and 49.28% for T45 networks, *P* < 0.001; Figure 3C). Interestingly, the PN density, which, at 1 DIV, was similar for all networks by experimental design (1,614.0 ± 30.8 cells/mm^2^; *n* = 210 cultures from nine experiments; Figure 3A, black bars), was systematically different among network types at 7 DIV: 1,308.9 ± 42.5, *n* = 149; T05: 1,071.89 ± 32.1, *n* = 150; T25: 718.3 ± 21.4, *n* = 240; T45: 548.3 ± 28.9, *n* = 113, mean ± SE (*P* ≤ 0.001; Figure 3B). Compared with the live counts at 1 DIV, cell loss was lowest in T00 networks and highest in T45 networks (Figure 3B).

Two different scenarios could explain this outcome. Due to the experimental design, the intended total neuron density was lowest in T00 networks and increased with every network type to the highest values in T45 (Figure 3A). If nutritional factors were limited in the culture medium, cell survival could potentially depend on total neuron density. Cell death rates would increase with the increasing number of cells resulting in cell density distributions as shown in Figure 3B. An alternative possibility would be that culture conditions were always sufficient, but that the density of INs had a direct or indirect effect on the cell survival of PNs. Thus, PN survival would be best in T00 and worst in T45 networks with declining values in-between.

To test the two possibilities, we designed experiments where network pairs had the same PN/IN ratio but different absolute plating densities (Figure 3D). In the low-density plating set (T00_L_/T45_L_) the cell culture pairs started with the original T00 plating density of 1,500 cells/mm^2^ (Figure 3A). Here also the T45 networks were adjusted to a total target density of 1,500 cells/mm^2^. In the high-density plating scheme (T00_H_/T45_H_; Figure 3D) culture pairs started with the original total plating density of the T45 networks (see Figure 3A) i.e., 2,800 cells/mm^2^. If the cell survival depends on total neuron density at plating, both, the high-density T00_H_ and the high-density T45_H_ should show a significantly higher cell loss rate compared with their low-density counterparts. If, however, the GABA cell content modifies PN cell survival, then the cell loss in high and low-density T45 networks should be higher than in the T00 counterparts.

The cell densities obtained in the live counts directly before adding MGE cells was as follows: The wild-type networks T00_L_ and T45_H_ had 1,587.9 ± 32.7 cells/mm^2^, T00_H_ had 2,856.3 ± 51.8 cells/mm^2^, T45_L_ had 1,014.8 ± 24.3 cells/mm^2^ (mean ± SE, in each set *n* = 150 fields from 5 experiments; Figure 3D, black bars). Based on these 1 DIV live cell counts T45_H_ and T45_L_ networks received the required number of MGE-INs to match cell densities of respectively T00H and T00L networks (Figure 3D, gray bars). After 1 week in culture the PNs densities (Figure 3E) were as follows: T00_L_: 1,482.7 ± 37.0 (*n* = 151); T00_H_: 2,402.8 ± 40.1 (*n* = 150); T45_L_: 445.0 ± 24.1 (*n* = 139); T45_H_: 582.0 ± 24.5 (*n* = 140). Thus, between 1 and 7 DIV in both T45 networks less than half of the originally counted PNs survived (43.8% in T45_L_ and 36.7% in T45_H_), while in both sets of T00 networks the PN survival was much higher (93.4%, in T00_L_; 84.1% in T00_H_; Figure 3E). To visualize the effects of IN content and total density, data of the four sets were normalized to their 1 DIV values, and then pooled in the following sets: T00 (T00H + T00L) and T45 (T45H + T45L), Low density (T00L + T45L) and High density (T00H + T45H). Figure 3F shows that the difference in total cell density had a smaller effect on PN density (MW-RST; *P* = 0.027, Low: *n* = 290, High: *n* = 290) than the difference in the IN content (MW-RST; *P* ≤ 0.001, T00: *n* = 301; T45: *n* = 379). No interaction was detected between the effects of network type and total density (*P* = 0.601, 2-way ANOVA).

The results above strongly suggest that IN density had an impact on PN survival. To address this possibility, we first investigated the occurrence of apoptosis during the 1st week in culture. Between 2–6 DIV the caspase expression was assessed with immunocytochemistry in 24 h intervals (T25 networks; Figures 4A–C,G). The number of apoptotic cells was very low at 2 DIV, increased dramatically by 3 DIV, and declined after that to a low level (*P* ≤ 0.001; KW-ANOVA). The steep increase in cell death was most probably due to the addition of the mitotic inhibitor Ara-C at 2 DIV. Since the Ara-C treatment induces apoptotic cell death during cell division (Banker and Goslin, 1991), we asked next if INs density might influence PNs proliferation rates in this initial phase of network development.

The proliferation rate of PNs and INs was investigated by BrdU immunocytochemistry 48–72 h after MGE-INs plating (Figures 4D–F,H,I). In IN depleted T00 networks, 68.3% of PNs were marked by BrdU. With increasing IN content, PN proliferation rates gradually increased to 73.2% in T05, 80.6% in T25, and 83.5% in T45 (*P* ≤ 0.001; chi-square-test; Figure 4H). Even the low IN content in T05 networks induced a significant increase in proliferation rates compared with the IN depleted T00 networks. These results suggest that the presence of INs has an activating effect on the proliferation rate of the PN population. These results may also explain why the 7 DIV density of PNs varies among the different network types (Figure 3B, black bars). Since Ara-C leads to apoptosis in dividing cells PN elimination is inevitably highest in T45 and lowest in T00 networks. INs showed considerably lower proliferation rates (Figure 4I) with no significant difference between T05 and T25 networks. However, proliferation increased in T45 networks compared with the other network types (Figure 4I). For the post-mitotic INs that migrate into the cortex, this aspect may be irrelevant. Whether the increase of proliferation rates in high GABA density is relevant for adjusting cell proliferation within the MGE remains to be shown.

### Long-Term Development of MGE-dCtx Networks

The quantitative analysis of the cell density development of PNs and INs after 7 DIV showed that cell elimination occurred in both neuron types with similar dynamics (Figure 5). The neuronal loss, substantial in the 2nd week (7–14 DIV), slowed down during the 3rd and 4th weeks in culture (14–28 DIV). Although INs content varied widely among different networks sets at 7 DIV [0.07% ± 0.02 (*n* = 149 fields) in T00, T05: 5.1% ± 0.3 (*n* = 149); T25: 24.4% ± 0.7 (*n* = 240); T45: 49.3% ± 1.6 (*n* = 113), and 90.6% ± 0.7 (*n* = 40) in MGE networks; Figure 5F], the fraction of INs within each set remained surprisingly constant over the 3 weeks, roughly reflecting the initial plating fraction. While there were no age differences in T00 (*P* = 0.127, KW-ANOVA) and T25 (*P* = 0.086, KW-ANOVA), the fraction of INs in T05 increased slightly (*P* = 0.004, KW-ANOVA). In T45 networks the IN fraction increased at 14 DIV (*P* = 0.005; Holm-Sidak), returning afterward to values similar to 7 DIV. The fraction of INs in MGE cultures (in the absence of cortical neurons), showed a small decline over age (*P* ≤ 0.001; KW-ANOVA; Figure 5F).

To compare the developmental dynamic of T05, T25, and T45 networks, we normalized their cell density values to the respective 7 DIV densities (Figures 5G–I). The normalized data showed that PNs followed a comparable decline in different network types (Figure 5G). Although PN density in T45 networks declined slightly faster between 14 and 21 DIV compared with T05 and T25 (*P* ≤ 0.001; KW-ANOVA), there was no difference among network types at 28 DIV (*P* = 0.062; KW-ANOVA). The normalized density values showed a better survival of INs in T05 networks (*P* = 0.012 at 14 DIV; *P* ≤ 0.001 at 21 and 28 DIV; KW-ANOVA; Figure 5H). IN densities differed between T25 and T45 networks only at 21 DIV (*P* ≤ 0.001; KW-ANOVA). Figure 5I shows the normalized fraction of INs, illustrating that between 7 and 28 DIV the fraction of INs remained stable in T25 and T45, but increased in T05 networks.

### Soma Size

Two observations lead us to measure the soma size of INs and PNs in different network types. First, in T05 networks many INs had larger cell bodies and more prominent dendrites and axons (Figure 5A) compared to their counterparts in T25 and T45 networks (Figures 5B,C), suggesting that INs in a low-density environment might develop larger dendritic trees and longer, more ramified axons. Second, the loss of INs over time was lower in T05 compared with other networks (Figure 5H), and the IN fraction increased with time in T05, but not in T25 or T45 (Figure 5I). Thus, in networks with very low IN density, the IN size and survival might increase to compensate for the deficit in numbers. The soma size analysis confirmed that INs in T05 networks differed from those in T20 and T30 networks (7 DIV: *P* = 0.010; 14 DIV: *P* < 0.001; and 21 DIV: *P* < 0.001; KW-ANOVA). Already at 7 DIV, IN somata were slightly larger in T05 compared with T20 and T30 networks (T20: *P* = 0.002; T30: *P* = 0.045; MW-RST; Figure 4J). This size difference increased at 14 (*P* < 0.001, MW-RST) and 21 DIV (*P* < 0.001; MW-RST). The size difference between INs of T20 and T30, detected at 14 DIV (*P* < 0.001; MW-RST), vanished at 21 DIV (*P* = 0.077; MW-RST). PNs did not show size differences among network types (*P* = 0.794, KW-ANOVA; Figure 4K). From these data we concluded that in low IN density each IN is likely to innervate more PN neurons.

### Long-Term Developmental Dynamics in MGE-wCtx Networks

In the experiments described above, we chose dissociated cells of dCtx to build T00 networks, because this part of the developing rat cortex does not contain migrating INs at E16. This allowed generation of networks that contain none or only very few isolated INs (0.07% ± 0.02, *n* = 149 fields, 7 DIV; Figure 6A, dCtx). In contrast, a wCtx preparation contained a small fraction of early-born INs (1.3% ± 0.1, *n* = 127 fields, 7 DIV; Figure 6A, wCtx), which had already migrated into the lateral portion of the E16 rat cortex. Most of these INs develop to large basket-like INs (L-GABA, see De Lima and Voigt, 1997; Voigt et al., 2001). Due to the developmental gradient, the wCtx preparation might also contain a higher fraction of post-mitotic projection neurons. This slightly more complex network showed a different developmental dynamic when cultivated with MGE-INs (Figures 6B–D, open circles). While in dCtx the fraction of INs increased or remained unchanged (Figures 5I, 6B–D, black dots), in wCtx networks the fraction of INs declined in all network types (*P* < 0.001 for T05, T25, and T45, KW-ANOVA; 6B–D, open circles, and Figure 6G). This was mainly due to a higher decline in the IN density in wCtx networks (Figures 6E,F) compared with the dCtx networks (Figures 5G,H). Interestingly, the faster decline in the IN fraction in wCtx (Figure 6G) did not occur if cultures were grown in gabazine, a GABA_A_ receptor antagonist (Figure 6H).

### IN Ratio Variation Effect on Network Activity

Since the results of population development analysis showed that MGE-dCtx networks could maintain a wide range of IN ratios for several weeks, we asked next if these networks differ in their physiology. To address this question, we first asked if network activity, as measured by Fluo-4 calcium imaging, differed when networks were constructed with low, medium, and high IN ratios (T05, T25, T45; Figure 7). As in the embryonic cortex, neurons in early neuronal cultures express a highly synchronized synaptic activity that leads to strong calcium influx into the cell (Opitz et al., 2002). GABA_A_R activity has been shown to have a dramatic effect on some parameters of spontaneous network activity (Baltz et al., 2010). Here we quantified burst frequency and burst attendance to compare different network types.

The cumulative distribution frequency (CDF) plots show that the frequency of the network bursts increased in networks with higher IN content (T05: 4 bursts/min, *n* = 82 fields; T25: 6.3 bursts/min, *n* = 90; and T45: 15.7 bursts/min, *n* = 87, median, *P* ≤ 0.001, KW-ANOVA; Figure 7A). Data of single cell frequencies confirmed this observation (T05: 4 bursts/min, *n* = 7807 neurons; T25: 5.7 bursts/min, *n* = 5674; T45: 9.5 bursts/min, *n* = 5256, median, *P* ≤ 0.001; Figure 7B). In contrast, with increasing IN content the tendency of a cell to participate in a network burst decreased: the median of the neuronal burst participation dropped from 100.0% in T05 (*n* = 1,538 burst events) to 94.0% in T25 (*n* = 1,538) and 72.2% in T45 (*n* = 1,538; median, *P* ≤ 0.001; Figure 7C).

The network activity recordings of 28-day-old cultures are illustrated in Figures 7D–F; see also Supplementary Material). T05 networks showed a stereotypic bursting behavior with 100% of all neurons participating in most bursts, which were each followed by a longer inter-burst interval with no activity. This participation pattern is typical for immature networks when GABA is depolarizing, or for older networks with insufficient GABAergic inhibition (Baltz et al., 2010). With increasing IN content, the network activity participation pattern showed less synchronization (Figures 7D–F). CDF plots for the recorded field show that with increased IN proportion, the burst participation dropped, i.e., fewer neurons were active together during most burst events (Figures 7E,F, right side).

Taken together, calcium imaging shows that although different PN/IN ratios could form functionally active networks, their activity patterns differed considerably. Networks built with a very low proportion of INs showed a stereotypical pattern of highly synchronized bursts, while networks with higher IN ratios showed a more heterogeneous pattern with an increased frequency of smaller burst transients with fewer cells participating.

### IN Ratio Variation Effect on Synaptic Transmission

**Figure 9:**
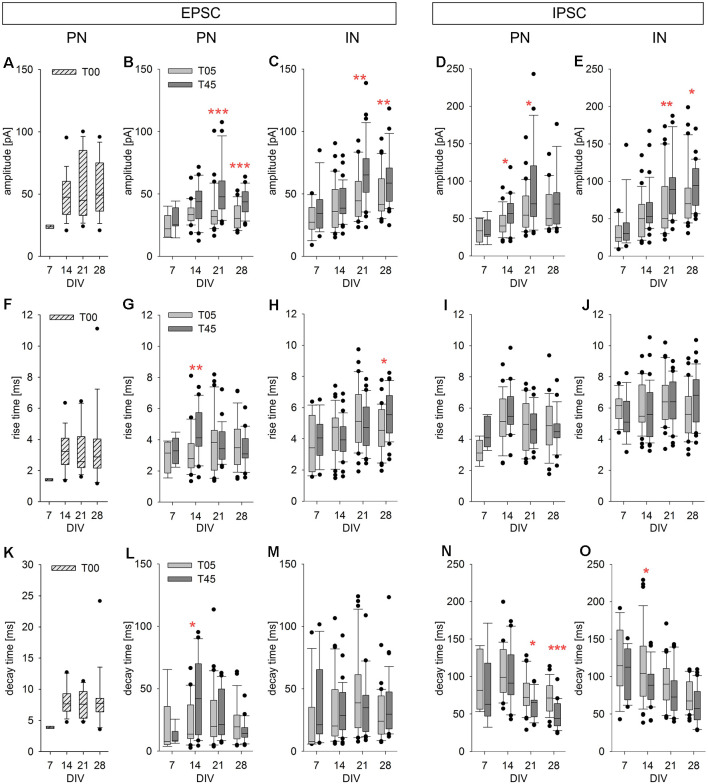
Developmental changes of isolated spontaneous PSCs in PNs and INs. The spontaneous postsynaptic currents analyzed for this and the next figure (see also Table 2) were isolated events during the interburst interval (see examples in Figure 8D, arrows). The box plots show the developmental changes of PSC amplitude **(A–E)**, rise time **(F–J)**, and decay time **(K–O)** for EPSCs [PNs (T00: **A,F,K**; T05 and T45: **B,G,L**); INs (**C,H,M**)] and IPSCs [PNs (**D,I,N**); INs (**E,J,O**)]. T00: hatched boxes; T05: light gray boxes; T45: dark gray boxes. Asterisks indicate significant differences between network types (*P ≤ 0.05, **P ≤ 0.01, ***P ≤ 0.001). For the number of cells and statistically significant age differences, see Table 2.

**Figure 10:**
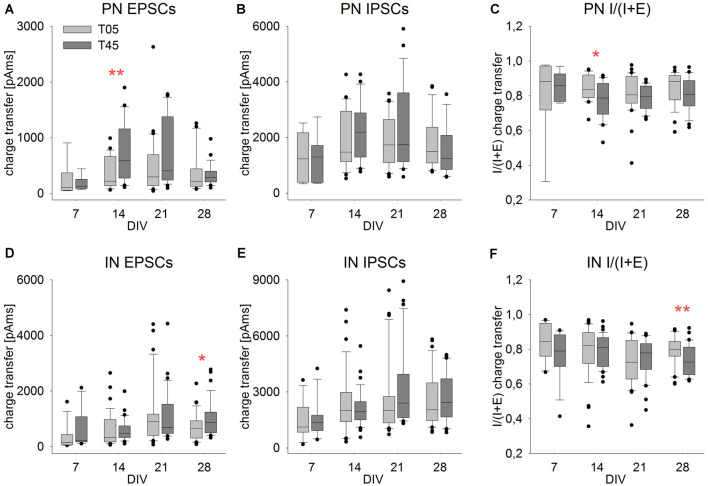
Isolated PSCs charge transfer ratio showed little variation in development and between networks. Graphs show the development of the single events PSC charge transfer and charge transfer ratio [I/(I + E)] in PNs **(A–C)** and INs **(D–F)** in T05 (light gray boxes) and T45 (dark gray boxes) networks over time. Compared with network bursts **(**Figures 8G–L**)**, single PSCs charge transfer differed considerably less over age and between network types. Asterisks indicate significant differences between network types (*P ≤ 0.05, **P ≤ 0.01). For the number of cells and statistically significant age differences see Table 2.

In additional experiments, we used patch-clamp recordings to measure the spontaneous glutamatergic and GABAergic postsynaptic currents (PSCs) in PNs and INs in T05 and T45 dCtx cultures. Figure 8 shows examples of the recorded activity and the analysis of large multisynaptic bursts. Figures 9, 10 and Table 2 show a summary of the analysis of isolated PSC events.

**Table 2:** Summary of EPSCs and IPSCs parameters in Projection Neurons (PNs) and GABAergic Interneurons (INs) of T00, T05, and T45 networks.

	PN								IN				
	T00		T05		T45				T05			T45	
DIV	*n*	Median	*n*	Median	*n*	Median	*P*^&^	*P*^$^	*n*	Median	*n*	Median	*P*^&^
**EPSCs**													
**Amplitude (pA)**													
7–9	2	23.77	6	22.10	7	25.23	0.366	0.510	12	27.28	17	34.38	0.438
12–16	17	45.50	24	33.73	20	43.85	0.106	0.024	39	35.98	35	38.69*	0.147
19–23	15	44.99	30	31.89	26	47.69*	<0.001	<0.001	30	44.53*	31	65.19	0.002
26–30	17	49.21	29	30.42	25	43.79	<0.001	<0.001	32	41.67	33	58.81*	0.009
P*		0.200		0.096		0.009				0.001		<0.001	
**Rise Time (ms)**
7–9		1.41		3.16		3.30	0.534	0.072		3.43		4.05	0.642
12–16		3.24		2.79		4.13	0.004	0.006		4.75		3.93	0.299
19–23		2.58		3.83		3.43	0.915	0.334		5.13*		4.75	0.323
26–30		2.88		3.50		3.10*	0.716	0.505		4.55		5.54*	0.015
P*		0.238		0.450		0.027				0.016		<0.001
**Decay (ms)**
7–9		3.89		7.67		8.26	0.945	<0.123		7.84		21.24	0.073
12–16		7.65		13.61		41.97*	0.024	<0.001		20.21		28.64	0.369
19–23		7.61		19.86		21.30	0.712	<0.001		38.82*		34.85	0.302
26–30		7.74		19.43		13.96*	0.306	<0.001		23.99		29.78	0.273
P*		0.172		0.425		0.002				0.010		0.869	
**Charge Transfer (pAms)**												
7–9		45.09		109.74		119.69	0.731	0.114		149.09		222.43	0.088
12–16		177.94		218.74		585.54*	0.010	0.003		325.32		475.67	0.330
19–23		230.71		294.97		405.62	0.086	0.014		892.94*		680.43*	0.629
26–30		178.95		218.24		289.70	0.245	0.208		640.78		867.99	0.039
P*		0.140		0.225		0.001				0.001		<0.001	
**IPSCs**													
**Amplitude (pA)**													
7–9			6	33.77	7	28.68	1.000		12	24.64	17	30.31	0.492
12–16			24	40.34	20	56.77	0.024		39	50.06	35	53.08	0.083
19–23			30	59.69	26	77.29*	0.015		30	50.43*	31	89.11*	0.006
26–30			29	49.92	25	69.48	0.331		32	70.16	33	94.39	0.015
P*				0.005		0.003				<0.001		<0.001	
**Rise Time (ms)**													
7–9				3.13		4.10	0.051			6.15		5.09	0.127
12–16				5.14		5.47	0.234			5.47		5.58	0.417
19–23				4.96		4.60	0.749			6.40		6.40	0.790
26–30				4.87		4.49	0.381			5.59		6.82	0.051
P*				0.054		0.005				0.226		0.039	
**Decay (ms)**														
7–9				81.73		63.02	0.731			114.58		112.58	0.438
12–16				98.92		91.26	0.823			104.12		88.47	0.049
19–23				72.00*		65.28*	0.026			89.88		72.68*	0.197
26–30				71.18		44.15	<0.001			67.44		57.39*	0.055

P*				<0.001		<0.001				<0.001		<0.001	
**Charge Transfer (pAms)**												
7–9				1230.41		1297.07	1.000			1117.23		1356.92	0.674
12–16				1474.04		2192.20	0.263			2002.62		1934.03	0.974
19–23				1731.17		1748.05	0.379			1997.69		2403.91*	0.102
26–30				1503.81		1240.23	0.160			2042.30		2432.01	0.315
P*				0.620		0.034				0.118		<0.001	
**I/(E + I)**													
**E/I Charge Transfer**												
7–9			6	0.88	7	0.86	0.534		12	0.85	17	0.79	0.163
12–16			24	0.84	20	0.79	0.018		39	0.82	35	0.81	0.289
19–23			30	0.81	26	0.80	0.186		30	0.73*	31	0.78	0.375
26–30			29	0.88	25	0.81	0.071		32	0.80	33	0.73	0.010
P*				0.389		0.223				0.012		0.118	

DIV, Days *in vitro*; *n*, number of neurons examined at each age and network set; P* = *P*-values from ANOVA or Kruskal-Wallis-ANOVA (KW-ANOVA), comparing different ages. P^&^ = P-values from Mann Whitney rank sum test (MW-RST) comparing the data from networks T05 and T45; *P^\^* = *P*-values from KW-ANOVA comparing the data from networks T00 with data from networks T05 and T45. The results of the multiple comparison tests for age differences (Holm-Sidak or Dunn’s Tests, *P* < 0.05) are indicated with an asterisk after the median, if this parameter at that age was significantly different from the one of the anterior age set (or more than one set, when these were not significantly different).

Before 9 DIV not all neurons received sufficient synaptic inputs to fulfill our criteria for a connected neuron (IPSCs and EPSCs ≥ 30). Some neurons had no or only very few PSCs, while others lacked one of both types. The total number of neurons that met the criteria in the time window of 6–9 DIV was as follows: PN in T05: 17.6% (*n* = 34); PN in T45: 33.8% (*n* = 21); IN in T05: 46.2% (*n* = 26) and IN in T45: 58.6% (*n* = 29). Although the fraction of connected neurons was lower in T05 networks compared with T45 for both cell types, the differences were not significant. If INs and PNs from both network types were pooled, more INs were connected (INs: 52.7%; PNs: 23.6%; *n* = 55; chi-square-test, *P* = 0.003). All neurons included in the following analysis fulfilled our criteria of connected neurons.

Spontaneous current recordings in INs and PNs consisted of single EPSCs and IPSCs (Figures 8A–C, arrows in **D**), short multiple synaptic events (Figure 8D, asterisk), and large network bursts. The latter had a peak amplitude of up to 800 pA, and duration in the second range (Figures 8D–F). They correspond to the network bursts recorded with calcium imaging (Opitz et al., 2002). We focused our analysis on large network bursts (Figures 8D–L) and on isolated spontaneous single EPSCs and IPSCs (Figures 8A–C, arrows in **D**, Figures 9, 10).

### sPSC Burst Events

Synchronous bursts of spontaneous glutamatergic (E) or GABAergic (I) activity appeared at low frequency as large inwardly directed (EPSC bursts) or outwardly directed (IPSC bursts) barrages of synaptic currents (Figures 8D–F). As a complement to the analysis of the imaging bursts, we calculated the charge transfer of bursts in individual PN and IN neurons of T05 and T45 networks from 12 to 30 DIV. The numbers of neurons analyzed at age groups 12–16 DIV, 19–23 DIV and 26–30 DIV were: T05 networks: PNs = 23, 27, 26; INs = 17, 31, 25; T45 networks: PNs = 19, 25, 26; INs = 21, 38, 40 neurons, respectively. Results are illustrated in Figures 8G–L.

EPSC burst charge transfer increased in PNs and INs in the 3rd week in comparison with the 2nd week, both in T05 and in T45 networks (in all cases, *P* < 0.001; Figures 8G,J). The burst charge transfer of EPSCs decreased in T45 in comparison with T05 [PNs: 2nd (*P* < 0.001) and 3rd week (*P* < 0.001), Figure 8G; INs: 3rd (*P* < 0.001) and 4th week (*P* = 0.002); Figure 8J].

IPSC burst charge transfer increased in the 4th week in PNs, but only in T45 networks (*P* = 0.02), in T05 age variations were not significant (*P* = 0.086; Figure 8H). The burst IPSC charge transfer was larger in PNs of T45 networks compared with those of T05 networks, but only in the 4th week (*P* = 0.043, MW-RST; Figure 8H). In INs, IPSC burst charge transfer increased in the 3rd week in T05 and T45 (in both cases, *P* < 0.001; Figure 8K). IPSC burst charge transfer in INs did not differ between T05 and T45 networks (Figure 8K).

To estimate changes in the balance of glutamatergic and GABAergic synaptic inputs, we calculated burst charge transfer ratios (E/I balance) for each cell from averaged values: GABAergic IPSC charge transfer/(IPSC charge transfer + EPSC charge transfer).

In PNs of T05 networks, the E/I ratio of network burst charge transfer was <0.5 and did not show age variation [2nd week: E/I = 0.47 ± 0.04 (mean ± SEM), *n* = 23; 3rd week = 0.35 ± 0.04, *n* = 27; 4th week = 0.42 ± 0.05, *n* = 26; *P* = 0.092, one-way ANOVA; Figure 8I, light gray boxes]. In PNs of T45 networks, E/I charge transfer ratio was ≥ 0.5 [2nd week: E/I = 0.62 ± 0.44 (mean ± SEM), *n* = 19; 3rd week = 0.62 ± 0.03, *n* = 25; 4th week = 0.50 ± 0.04, *n* = 26; *P* = 0.031, ANOVA; Figure 8I, dark gray boxes].

In INs, the E/I ratio of network burst charge transfer showed age variation only in T05 networks [2nd week: E/I = 0.54 ± 0.06 (mean ± SEM), *n* = 17; 3rd week = 0.31 ± 0.04, *n* = 31; 4th week = 0.41 ± 0.04, *n* = 25; *P* = 0.002, one-way ANOVA; Figure 8L, light gray boxes]. In T45, the E/I charge transfer ratio was > 0.5 with no significant age variation [2nd week: E/I = 0.59 ± 0.04 (mean ± SEM), *n* = 21; 3rd week = 0.55 ± 0.03, *n* = 38; 4th week = 0.57 ± 0.02, *n* = 40; *P* = 0.634, one-way ANOVA; Figure 8L, dark gray boxes].

The multisynaptic network bursts E/I ratio was significantly reduced in PNs and INs of T05 networks compared with T45 networks [PNs: 2nd (*P* = 0.012) and 3rd week (*P* < 0.001), Figure 8I; INs: 3rd (*P* < 0.001) and 4th week (*P* < 0.001); Figure 8L]. Thus, during the burst of synaptic activity, the inhibition provided by the low density of INs in T05 networks did not counterbalance the excess of excitatory synaptic drive provided by the PN neurons’ synapses.

### sEPSC Single Events in T00 Networks

Because the first INs entering the embryonic cortex may innervate other neurons profusely even in low density (Voigt et al., 2001), and very rarely, isolated INs can be found in dCtx T00 networks (Figure 5E), we assessed, for control, IPSC inputs in PNs in T00 networks. Of the 65 PNs recorded between 6–30 DIV in T00, the majority (78.5%, *n* = 51) showed only EPSCs and not one IPSC during the 2 × 5-min spontaneous activity recordings. The remaining 14 neurons showed EPSCs (≥30) and at least 10 or more IPSCs. The results confirmed that in T05 and T45 networks the vast majority of GABAergic innervation was provided by the MGE-INs.

The development of EPSC parameters (amplitude, rise time, decay time, and charge transfer) for PN neurons of T00, T05, and T45 networks is shown in Table 2 and Figures 9A,B,F,G,K,L; data is aggregated in four age periods around 7, 14, 21, and 28 DIV. EPSC parameters did not change over age in T00 networks (Table 2).

EPSC amplitudes in PN of T00 networks showed moderately increased amplitudes when compared with T05 (*P* < 0.05, Dunn’s test). In contrast, decay times were markedly smaller in T00 than in T05 and T45 (in both cases, *P* < 0.05, Dunn’s test, Table 2). Charge transfer in T00 was significantly decreased in the 2nd and 3rd week only when compared with T45 (*P* < 0.05, Dunn’s test, Table 2).

### Development of sEPSCs Single Events in PNs and INs of T05 and T45 Networks

An age-dependent variation in the EPSC parameters (amplitude, rise time, and decay time) was detected in PNs only in T45 networks (Table 2, Figures 9B,G,L, dark gray boxes). In contrast, IN EPSCs showed significant age variations in both T05 and T45 networks (Table 2; Figures 9C,H,M).

EPSCs amplitude increased with development in T45 in both neuron types; in T05 a significant increase was detected only in INs. In the first 2 weeks, EPSCs recorded in T45 tended to be slightly larger than those in T05, but in the 3rd and 4th week, the amplitude was significantly larger in T45 networks compared with T05 in both PNs and INs (Table 2, Figures 9B,C). The rise time of EPSCs in PN (T45) was largest by the 2nd week, then decreased between the 2nd and 4th week (Figure 9G). In contrast, in INs, EPSCs rise time increased to the 3rd (T05) or 4th (T45) week *in vitro* (Figure 9H). Differences between network types reflected the diverse developmental dynamic in PN (T05 and T45 different in the 2nd week) and IN (T05 and T45 different in the 4th week; Table 2, Figures 9G,H). EPSCs decay time showed a maximal value in the 2nd week in PNs, decreasing in the following weeks (T45, Table 2, Figure 9L), INs showed a maximum value at the 3rd week (T05), but the small decrease afterward was not significant (Table 2, Figure 9M). The decay time of EPSCs differed between network types only in PNs (2nd week, Table 2, Figure 9L).

### Development of sIPSCs Single Events in PNs and INs of T05 and T45 Networks

All IPSC parameters showed significant age variation in T45 networks (Table 2, Figures 9D,E,I,J,N,O, dark gray boxes) of both neuron types. In T05 networks (light gray boxes) age variation was detected in amplitude (Figures 9D,E) and decay time (Figures 9N,O) in both PNs and INs. Amplitude increased until the 3rd week (T45 in PN and IN, T05 in IN; Table 2, Figures 9D,E). IPSCs rise time showed moderate variation in T45 networks: in PNs maximum value in the 2nd week; in IN, in the two last weeks (Table 2, Figures 9I,J). The decay time of IPSCs decreased after the 2nd week in both neurons and network types (Table 2, Figures 9N,O). Both amplitude and decay time of IPSCs showed significant differences between network types: Amplitude values were larger in T45 networks in both neuron types (PNs in the 2nd and 3rd weeks, INs in the 3rd and 4th weeks; Table 2 and Figures 9D,E). Decay times in neurons of T45 were shorter than in T05 networks (PNs: 3rd and 4th weeks, INs: the 2nd week; Table 2 and Figures 9N,O).

### Charge Transfer Balance Between Glutamatergic and GABAergic Synaptic Inputs

We compared the charge transfer of isolated EPSCs and IPSCs at different ages and networks to later estimate changes in the balance of glutamatergic and GABAergic synaptic inputs (Table 2, Figures 10A,B,D,E).

The EPSCs charge transfer (Table 2, Figures 10A,D) increased to a maximum in the 2nd week in PNs (T45) and in the 3rd week in INs (T05 and T45). These variations in EPSCs charge transfer reflected similar changes in amplitude and decay time in each neuron type (see above). The comparison between network types showed little variation (Figures 10A,D, Table 2): EPSC charge transfer was increased in T45 compared with T05 during the 2nd week in PNs (Table 2, Figure 10A) and in the 4th week in INs (Figure 10D). IPSCs charge transfer values increased in the 3rd week (T45 in INs, Table 2, Figures 10B,E) and were larger in INs than in PNs (2-way ANOVA, *P* = 0.022). In each neuron type, IPSCs charge transfer did not differ between T05 and T45 networks (Table 2, Figures 10B,E).

The charge transfer ratios (E/I balance) was calculated for each cell from averaged values: sIPSC charge transfer/(sIPSC charge transfer + sEPSC charge transfer). Independent of the number of MGE-INs in the network, the charge transfer of isolated IPSCs was always larger than the charge transfer of EPSCs (Figures 10A,B,D,E). As a consequence, the E/I ratio of the charge transfer was larger than 0.5, as was also described in mice cortical cultures (Klueva et al., 2008). The charge transfer ratio in PNs of both T05 and T45 networks and INs of T45 networks did not change over time (Table 2, Figures 10C–F). INs of T05 networks showed a slight decrease in charge transfer balance in the 3rd week (Table 2, Figure 10F). The E/I balance in PNs and INs did not differ between T05 and T45 (PN: *P* = 0.178; IN: *P* = 0.058, 2-way ANOVA). In isolated age sets, however, neurons in T45 showed a lower charge transfer ratio than in T05 networks: PNs in the 2nd week, and INs in the 4th week (Table 2, Figures 10C,F).

Taken together, the analysis of spontaneous synaptic current events showed that, whatever the PN/IN proportions in the network, the E/I balance in isolated PSCs was mostly constant. If, however, larger spontaneous multisynaptic events were considered (network bursts; Figure 8), significant differences of E/I balance were detected when comparing networks built with distinct PN/IN proportions.

## Discussion

By building cortical networks of dissociated progenitor neurons from the early cortical anlage of embryonic rats together with embryonic MGE-INs, we found that in the first days in culture the number of proliferating cortical PNs increased with the number of co-cultured INs (Figure 4H). This finding suggests that an initial regulatory step in setting up the cellular ratio between INs and PNs could be the ability of migrating INs to modulate the proliferation rate of PNs in their target area. A regulatory coupling between the number of ingrowing INs and the number of generated PNs would allow for an initial cell ratio adjustment on the basis of cell proliferation before activity-dependent cell elimination takes over at later developmental stages for fine adjustments.

INs migrate into the cortex in two horizontal streams, one in the intermediate zone and one in layer one (for review see (Jovanovic and Thomson, 2011). Particularly, those neurons migrating within the intermediate zone are in close vicinity to the proliferating progenitor cells in the ventricular zone below. It is well documented that growth cones of migrating INs constantly release GABA into their environment (Taylor et al., 1990; Taylor and Gordon-Weeks, 1991; Gao and Pol, 2000; Demarque et al., 2002). Proliferating neurons in the ventricular and later in the subventricular zone are, on the other hand, competent to respond to GABA and to glutamate. They express several GABA_A_ receptor isoforms (Laurie et al., 1992; Owens et al., 1999; Lujan et al., 2005) and at least one kainate receptor subunit of the glutamate receptor family (Herb et al., 1992; Lujan et al., 2005). Both amino acid neurotransmitters affect the cell cycle. Initially, it was found that both applied GABA and glutamate reduce cell proliferation rates (Loturco et al., 1995; Antonopoulos et al., 1997). Consecutive analyses showed however that ambient GABA increases the proliferation rate of progenitor cells in the early formed ventricular zone but decreases it in the later formed subventricular zone (Haydar et al., 2000). The reduction of proliferation can be triggered by intracellular Ca^2+^ increases after depolarizing tonic activation by GABA_A_ receptors (Liu et al., 2005; Bordey, 2007; Young et al., 2012). GABA_A_ receptors may also activate the S/G2 DNA-damage checkpoint pathway and inhibit cell cycle progression, as showed in embryonic stem cells (Andang et al., 2008; Wang and Kriegstein, 2009; Xing and Huttner, 2020). GABA-dependent increases in proliferation in the ventricular zone may be mediated by GABA_B_ or GABA_A_ receptors and the recruitment of different growth-stimulating factors, as was reported in an isolated mouse neural progenitor cells culture system from the mouse brain (Fukui et al., 2008a,b; Xing and Huttner, 2020).

At E16, the rat dCtx preparation contains predominantly proliferative early ventricular zone progenitor neurons. Modulation of the cell cycle within this cell population has a profound effect on the number of projection neurons generated for the individual layers (Takahashi et al., 1999). Cortical progenitor neurons of the lower cortical layers are born in the ventricular zone at a time when the first wave of INs migrates into the cortex. Thus, the link between ingrowing IN density, ambient released GABA from their growth cones, and GABA dependent cell cycle regulation of progenitor cells within the ventricular zone could adjust population ratios before any synapses are formed.

After the onset of synaptogenesis in cultured networks, the number of both PNs and INs declined due to activity-dependent apoptotic cell death (Verney et al., 2000; Voigt et al., 2001; Opitz et al., 2002). This is similar also in the timescale to the development in the intact brain and in cultures of other mammalian species (Southwell et al., 2012; Wong and Marin, 2019). In the dCtx cultures enriched with MGE progenitors, both PN and IN populations go through the process of cell elimination with the same dynamics (dCtx; Figures 5, 6), and the fraction of interneurons did not change over a 1-month cultivation period despite a wide range of ratios tested. On the other hand, if the MGE-INs were added to neurons from the entire cortex, the decline of the IN population was steeper than the decline of the PN population (wCtx; Figure 6). Interestingly, the resulting decline in the IN fraction could be blocked by GABAR_A_ antagonists (Figure 6H), suggesting a role for the GABA mediated neurotransmission in the INs long-term integration in the network.

The cellular structure of the dCtx and wCtx at the time of dissociation is relevant to understand the different outcomes of the cultures. In rats, the dorsal cortex consists at E16 mainly of the ventricular zone and layer 1. At this age, other cortical layers have not formed, and MGE-INs have not invaded the entire upper pole (see IN depleted T00 networks; Figures 1A, 5E), but the first cohort of early- born MGE-INs are already migrating in the lateral cortical anlage (Voigt et al., 2001). Besides the presence or absence of INs, the cortical tissue develops gradually from lateral to dorsal. While in the dorsal cortex future layer 6 neurons are still proliferating, lower cortical layers neurons are already post-mitotic in the most lateral cortex, and the formation of the upper layers has already begun (Berry and Rogers, 1965). Thus, at dissociation time the wCtx included the first cohort of early-born INs and a larger fraction of post-mitotic projection neurons. Birth dating studies have shown that this first population of INs is born in rats between E12 and E15. They include the population of INs involved in initiating and coordinating early network activity (Voigt et al., 2001). Since these neurons are an integral part of the functional subplate, the difference between dCtx and wCtx preparations also reflects the presence or absence of neurons that belong to this developmentally extremely important transient layer. It has been shown in many studies that throughout the cortical development the subplate serves diverse organizational functions (Kanold and Luhmann, 2010; Luhmann et al., 2018). This includes the early initiation of synchronized activity required for maturation and consolidation of synaptic connections (Voigt et al., 2005; Yang et al., 2009). In our cell cultures both dCtx and wCtx networks contained electrically active INs. However, ratio adjustment occurred only in the wCtx networks, which contained early-born INs. This strongly suggests that these neurons have a special function not only in the initiation of early synchronous network activity (Voigt et al., 2001; Opitz et al., 2002; Ben-Ari, 2007; Ben-Ari et al., 2007; Luhmann and Khazipov, 2018) but also in the adjustment of the PN/IN ratio.

Thus, the long–term culture results suggest that the PN/IN ratio regulation depends on the network structure at the developmental window when activity-dependent cell elimination takes place. When the developing cortex is immature (e.g., dCtx), the relative proportion of MGE-INs stays fairly constant. If, instead, the INs are added to a network that has reached critical structural maturity (e.g., wCtx), the observed decrease in the proportion of INs hints at an additional, at least partially GABA-dependent population adjustment mechanism.

In accordance with the results in cortical culture networks, when embryonic MGE-INs are transplanted in the postnatal mice neocortex (P3) or into the adult cortex, INs integrate successfully into the network (Southwell et al., 2010, 2012; Casalia et al., 2017; Priya et al., 2019). In the mouse cortex the PN/IN ratio was shown to be established early postnatal in a two-phase process (Wong et al., 2018). After an initial decline in the total number of PNs between P2 and P5, apoptosis occurs among the INs between P5 and P10. The latter process is activity-dependent and driven by the population of PNs. During the phase of IN selection, the required ratio is established by activity- dependent recruiting of well-connected INs into the electrically active network of PNs (Wong et al., 2018). Compared with the mouse, the rat cortex develops on a slightly prolonged time scale. Thus, apoptosis extends over a 3-week period in cultured rat cortex networks (developmental age P14 corresponds roughly to 21 DIV). In the present study, the network with higher IN density (T45) showed a hint of a similar sequential elimination process. Between 7 and 21 DIV, the elimination of PNs preceded that of INs in T45 networks (Figures 5G,H).

Summing up, the analysis of the neuronal population development showed that early structural differences in networks may be maintained beyond the activity-dependent cell elimination period. The question remains if the functional synaptic development mirrors the cellular structure or if subcellular rearrangements compensate the differences.

The calcium imaging analysis of network activity confirmed significant differences between network types (Figure 7). Increasing INs proportion in the cultured networks resulted in not only higher burst frequencies, but also an increase in bursts with low neuron participation (Figure 7). In cultured networks with very few INs, all neurons were active in all network events, a typical pattern for very immature networks, networks without INs, or networks with blocked GABA transmission (Opitz et al., 2002; Baltz et al., 2010). These large–scale network bursts (various seconds in duration and interburst intervals of up to minutes) appear at the end of the 1st week *in vitro* and achieve maximal attendance (percentage of the number of cells participating in the burst event) during the 2nd week *in vitro*. Although the early emergence of this stereotypic network pattern is facilitated by the depolarizing drive of GABAergic neurons (Ben-Ari et al., 1997; Ben-Ari, 2002; Opitz et al., 2002), these synchronous bursts are driven by ionotropic glutamatergic receptors.

In the 2nd and 3rd weeks *in vitro*, the frequency of burst activity increases, and the pattern of larger bursts is often enriched with bursts showing smaller amplitude and lower attendance, which disappear after application of the GABA_A_ antagonists. These smaller bursts are also absent in cultures without early L-GABAergic neurons, large neurons with long–range connections (Baltz et al., 2010; Baltz and Voigt, 2015). A further development of network activity is a temporal burst clustering that can be first observed by the end of the 3rd week *in vitro*. The activity clustering is characterized by alternating periods of higher and lower burst incidence (Baker et al., 2006; Wagenaar et al., 2006a,b; Baltz et al., 2010), and corresponds to slow changes of network excitability (Baltz and Voigt, 2015). In GABA neuron depleted dCtx cultures and in cultures with blocked GABA_A_ receptors this development is not observed, indicating that the slow changes of network excitability are dependent on GABAergic transmission (Baltz et al., 2010). Our results are consistent with earlier results from calcium imaging and MEA recordings showing a GABA-dependent development of complex activity patterns (Baltz et al., 2010, 2011; Baltz and Voigt, 2015; Haroush and Marom, 2019). Thus, the presence of sufficient GABAergic neurons not only contributes to the initial synchronization of network activity but is also essential for the later development of desynchronized patterns of network activity.

The whole-cell patch clamp analysis showed that the E/I balance for single PSCs did not change dramatically over time, across network types, or between INs and PNs (Figures 10C,F). This shows that PSC parameters may adjust over a wide range of IN ratios in the network and in different cell types despite the fact that they vary over age (Figure 9, Table 2). Various mechanisms, such as synaptic recruitment, regulation of intrinsic excitability, or alterations of membrane resting potential, could contribute to assure E/I stability subcellularly (Hartman et al., 2006; Isaacson and Scanziani, 2011; Chen et al., 2012; Xue et al., 2014; He and Cline, 2019). The increased soma size (Figure 4J) as well as the increased cell survival of INs in T05 networks (Figures 5F,I) indicate that morphological adaptation processes may also be involved.

In contrast to isolated PSC measurements, network burst currents reflect the coactivation of many neurons. In accordance with the calcium imaging analysis (Figure 7), the current clamp recording of multisynaptic PSC bursts show marked differences between T05 and T45 (Figure 8). Networks with lower IN density showed a decrease in the relative strength of inhibitory currents in single cells, IN, or PN (Figures 8I,L).

Taken together the electrophysiological analysis showed that the E/I balance for single synaptic events varies minimally over age and over a wide range of IN/PN ratios (Figures 9, 10). If multisynaptic network activity comes into play (Figures 7, 8) differences in interneuron content are reflected in different network behavior. Interneurons show a surprising ability to increase arborization when present in low density, but subcellular remodeling or synaptic plastic changes may not be sufficient to compensate for the scarcity of INs or the lack of specific types of INs during the early stages of network development. Even if the presence of few INs may assure cellular E/I balance, the network activity patterns reflect the structural deficiency.

## Data Availability Statement

The raw data supporting the conclusions of this article will be made available by the authors, without undue reservation.

## Author Contributions

Conceived and designed the experiments: AL and TV. Performed the experiments and analyzed the data: WX and TV. Wrote the article: WX, AL, and TV. All authors contributed to the article and approved the submitted version.

## Conflict of Interest

The authors declare that the research was conducted in the absence of any commercial or financial relationships that could be construed as a potential conflict of interest.
