# Supplementary figures and images for: The Structural E/I Balance Constrains the Early Development of Cortical Network Activity
Source: Front Cell Neurosci. 2021 Jul 19;15:687306. doi: 10.3389/fncel.2021.687306 (PMC8326976; doi:10.3389/fncel.2021.687306)

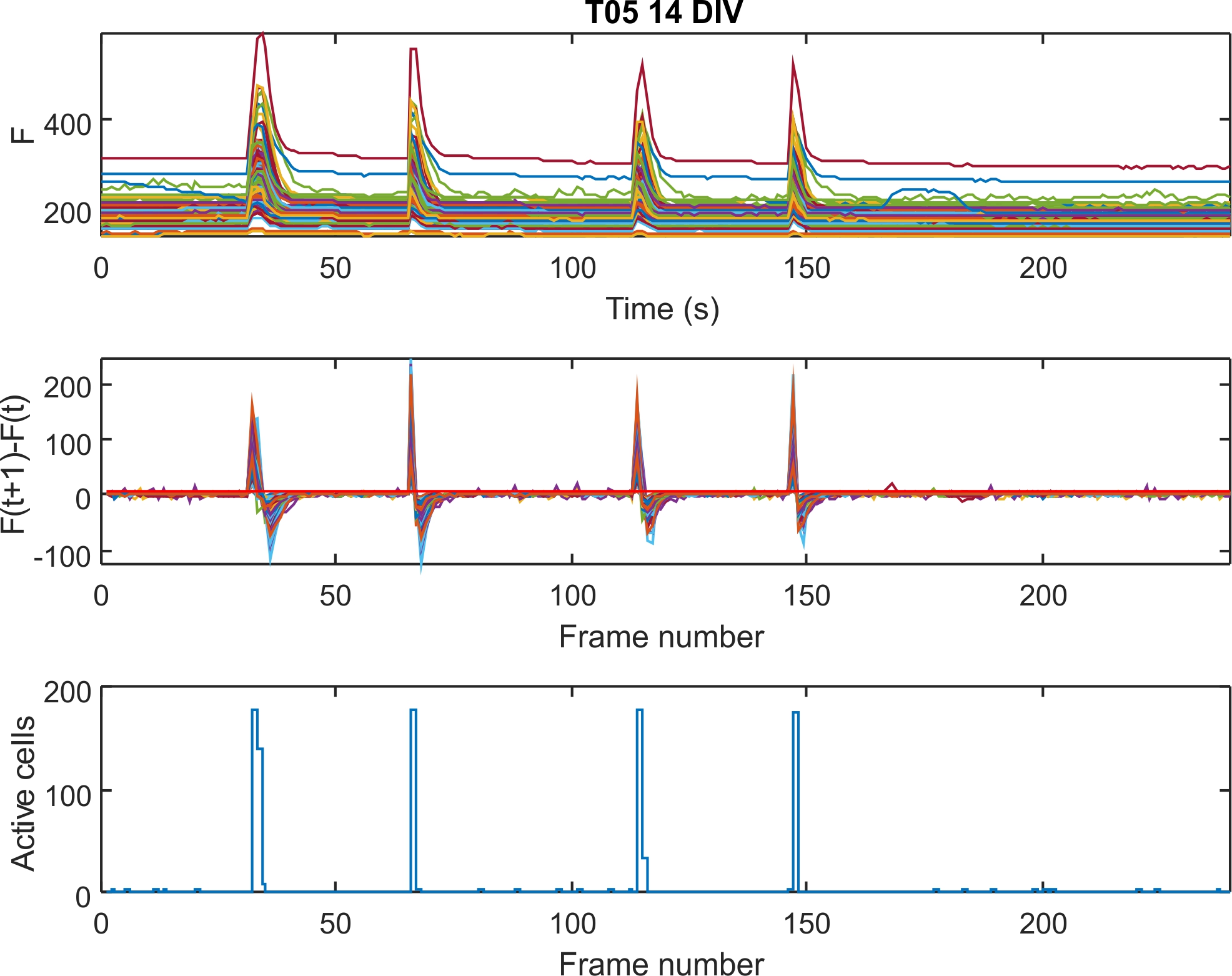

Supplement: Supplementary file 1 [file Image_1.jpg]

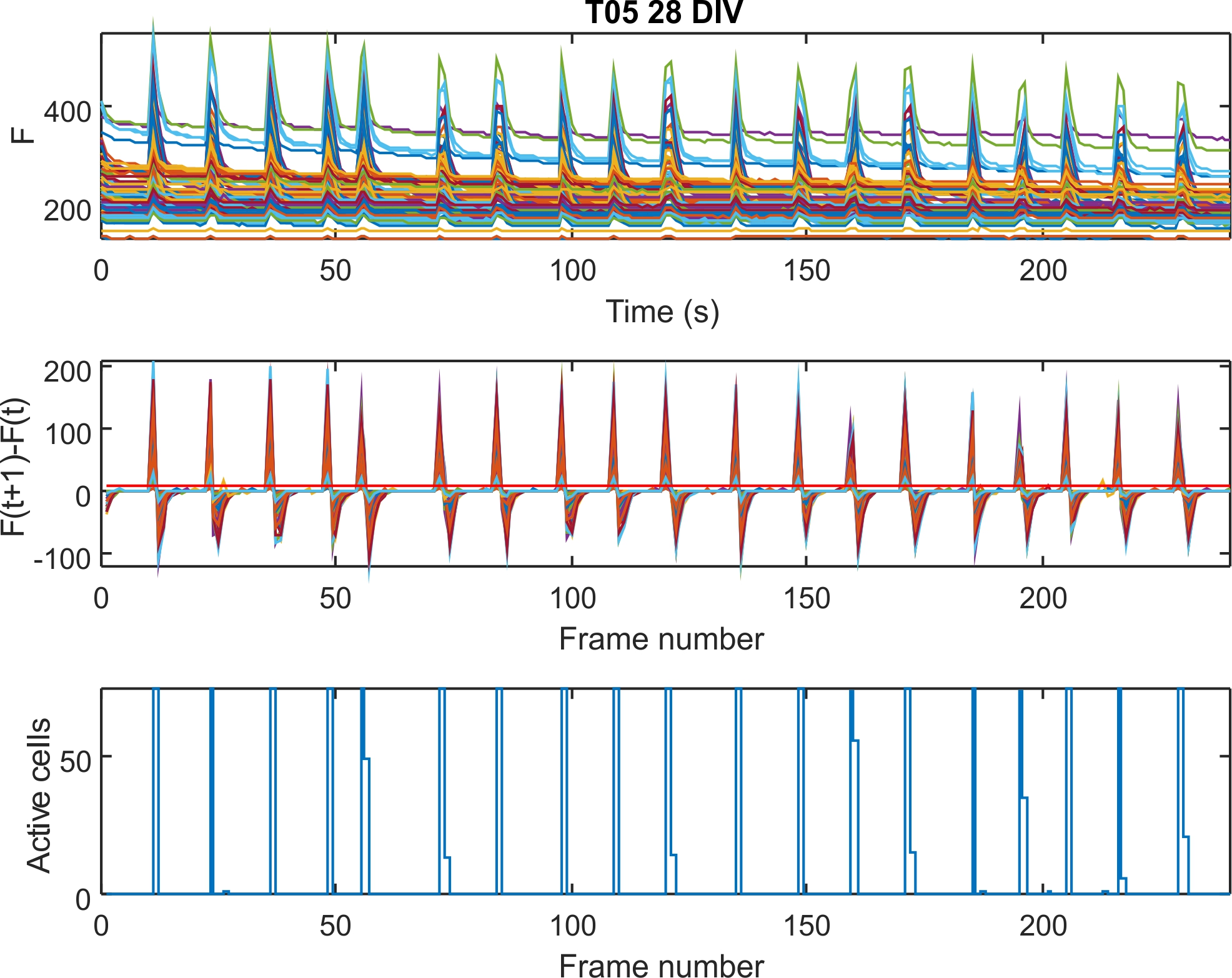

Supplement: Supplementary file 2 [file Image_2.jpg]

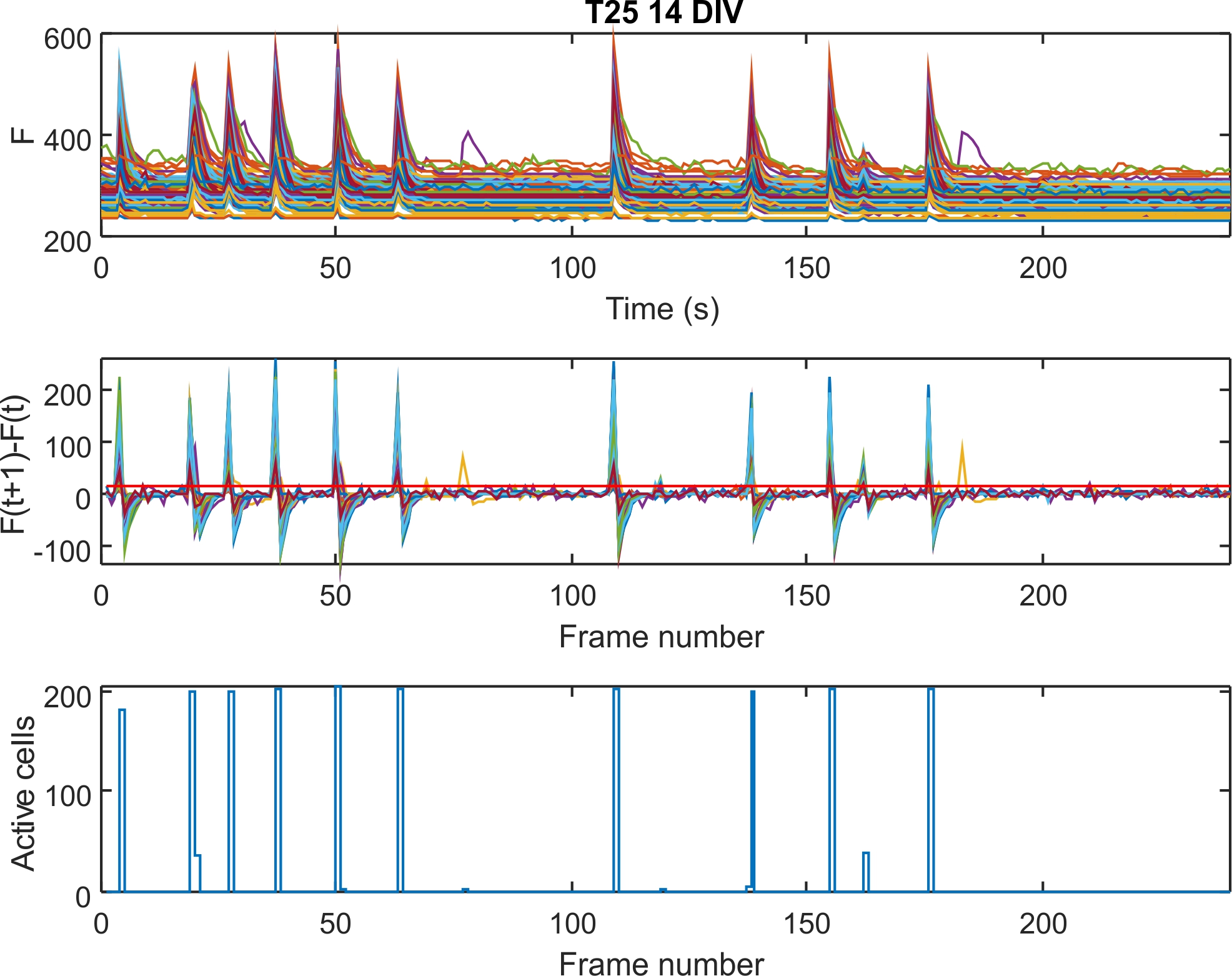

Supplement: Supplementary file 3 [file Image_3.jpg]

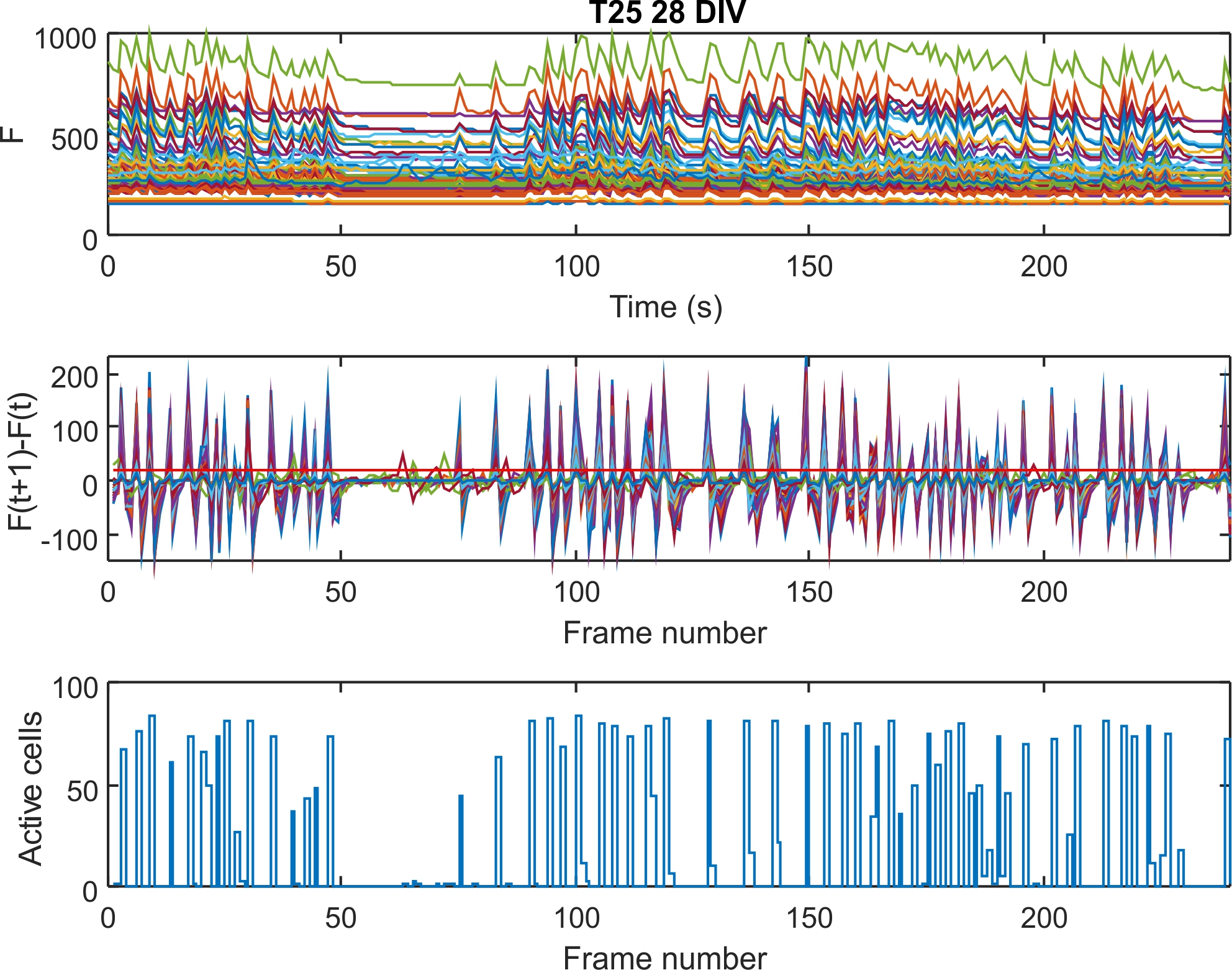

Supplement: Supplementary file 4 [file Image_4.jpg]

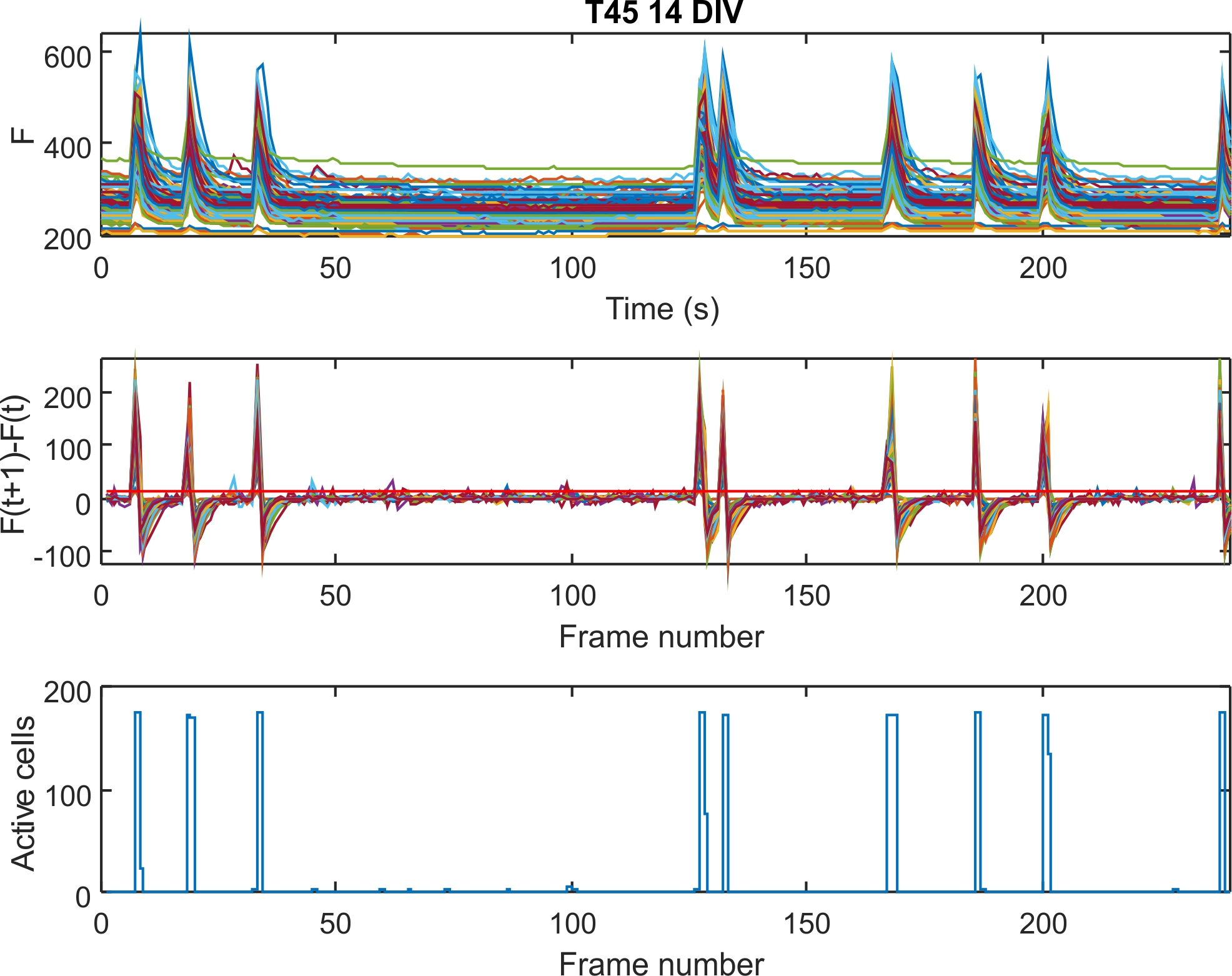

Supplement: Supplementary file 5 [file Image_5.jpg]

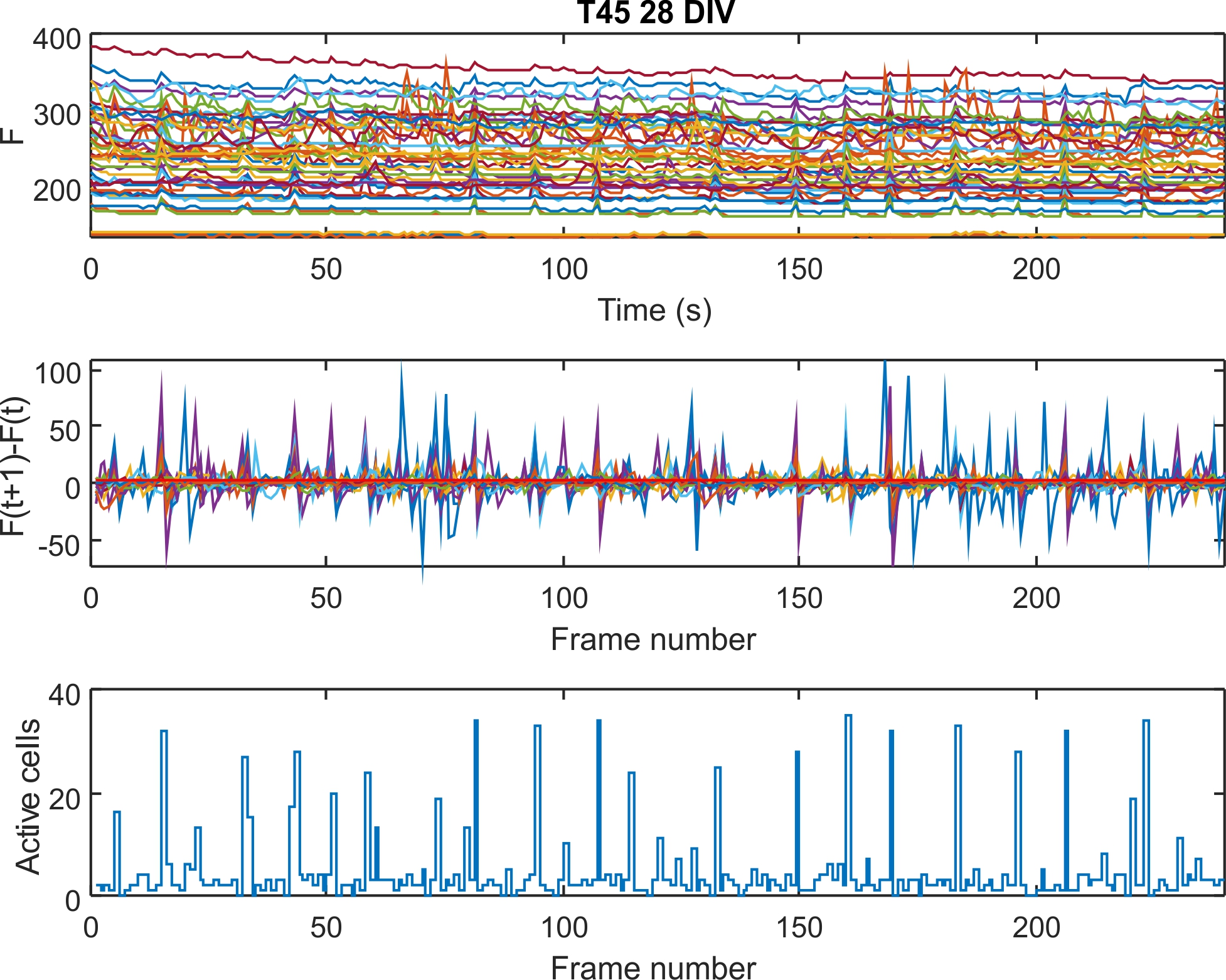

Supplement: Supplementary file 6 [file Image_6.jpg]
