# Supplementary material for: The Structural E/I Balance Constrains the Early Development of Cortical Network Activity
Source: Front Cell Neurosci. 2021 Jul 19;15:687306. doi: 10.3389/fncel.2021.687306 (PMC8326976; doi:10.3389/fncel.2021.687306)
Supplement: Supplementary file 13 [file Data_Sheet_1.docx]

Structural E/I Balance in Developing Cortex

Xing W, de Lima AD and Voigt T (2021)

Supplementary Material

Exemplary sequences of calcium imaging recordings.

The videos show exemplary fields of T05, T25 and T45 networks at 14 DIV and 28 DIV (see also figures 7 D, E, F).

The jpg-files show the MATLAB generated graphs for each video sequence, as follows:

The upper graph shows the unprocessed changes of fluorescence intensity (4 min recording period, 240 frames).

The middle graph shows F(t+1)-F(t) (see method). These traces are shown in the corresponding movies. The red line in the graph indicates the threshold that was calculated from three background ROIs (see method). If the fluorescence change in a neuron exceeded the threshold it was counted as active (see lower graph).

The lower graph shows the number of active neurons in each frame.

| JPG - File | MP4 - File | Network | Age |
| --- | --- | --- | --- |
| Image 1 | Video 1 | T05 | 14 DIV |
| Image 2 | Video 2 | T05 | 28 DIV |
| Image 3 | Video 3 | T25 | 14 DIV |
| Image 4 | Video 4 | T25 | 28 DIV |
| Image 5 | Video 5 | T45 | 14 DIV |
| Image 6 | Video 6 | T45 | 28 DIV |
